# Osteocalcin Beyond Bone: Molecular Mechanisms, Endocrine Networks, and Translational Perspectives Across Metabolism, Neurobiology, and Chronic Disease

**DOI:** 10.3390/ijms27072992

**Published:** 2026-03-25

**Authors:** Wiktor Derwich, Karolina Feć, Aleksander Gawda, Kamil Kopa, Jan Kopeć, Igor Nowak, Natalia Seńcio, Abdur Rauf, Zubair Ahmad, Alicja Świątek-Pawelczak, Dorota Formanowicz

**Affiliations:** 1 Faculty of Medicine, Poznan University of Medical Sciences, 60-812 Poznań, Poland; wiktor.derwich@gmail.com (W.D.); feckarolinam1@gmail.com (K.F.); alegawda@gmail.com (A.G.); kamil.kopa01@gmail.com (K.K.); j.kopec@macron.pl (J.K.); igor.nowak@icloud.com (I.N.); nataliasencio123@gmail.com (N.S.); 2 Department of Chemistry, University of Swabi, Swabi 23561, Khyber Pakhtunkhwa, Pakistan; mashaljcs@yahoo.com (A.R.); za3724364@gmail.com (Z.A.); 3 Chair and Department of Medical Chemistry and Laboratory Medicine, Poznan University of Medical Sciences, Rokietnicka 8, 61-701 Poznań, Poland; aswiatekpawelczak@ump.edu.pl

**Keywords:** osteocalcin, undercarboxylated osteocalcin, bone metabolism, endocrine signaling, GPRC6A/GPR158 pathways, vitamin K-dependent carboxylation, metabolic regulation

## Abstract

Osteocalcin (OCN) is increasingly recognized as a multifunctional hormone whose actions extend far beyond its traditional role as a marker of bone turnover. This review provides an integrated examination of the molecular, endocrine, and translational dimensions of osteocalcin biology, with emphasis on its bioactive undercarboxylated form (ucOCN), which links skeletal remodeling to systemic physiological processes. The structural determinants, biosynthetic pathways, and vitamin K-dependent carboxylation mechanisms underlying OCN isoform diversity are summarized, together with analytical limitations arising from assay variability and differences between N-MID and ucOCN-specific measurements. Mechanistic evidence demonstrates that ucOCN signals through GPRC6A and GPR158 to modulate insulin secretion, muscle glucose uptake, adipokine production, testosterone synthesis, neurocognitive function, hepatic lipid metabolism, and acute stress response. These receptor-level pathways position osteocalcin as a central regulator at the intersection of bone metabolism and whole-body homeostasis. The review synthesizes data across major clinical contexts, including metabolic syndrome, type 2 diabetes (T2DM), non-alcoholic fatty liver disease (NAFLD), chronic kidney disease–mineral and bone disorder (CKD-MBD), cardiovascular dysfunction, and neurodegeneration, highlighting the modifying influence of vitamin K status, circadian rhythms, renal clearance, and local tissue microenvironments. The need for biomarker standardization, methodological harmonization, and receptor-targeted translational strategies is emphasized, alongside emerging therapeutic concepts involving vitamin K supplementation and exercise-induced activation of OCN. Collectively, the evidence reframes osteocalcin as a versatile endocrine mediator at the interface of bone physiology, systemic metabolic regulation, and disease mechanisms.

## 1. Introduction

Bone is increasingly recognized as an endocrine organ, and osteocalcin (OCN) exemplifies this paradigm shift. Initially regarded as a marker of bone turnover, OCN is now known to exert systemic effects on metabolism, fertility, the nervous system, and stress physiology. These actions are largely attributed to its undercarboxylated form (undercarboxylated osteocalcin (ucOCN)), which circulates as a bioactive hormone and signals through several G protein-coupled receptors, including G protein-coupled receptor class C group 6A (GPRC6A), G protein-coupled receptor 158 (GPR158), and G protein-coupled receptor 37 (GPR37). Through these pathways, OCN links skeletal remodeling with glucose homeostasis, muscular energy adaptation, male reproductive function, neurocognitive processes, and hepatic metabolism.

Despite rapid progress, current knowledge on OCN remains fragmented. Evidence arises simultaneously from metabolic research, neuroendocrinology, hepatology, reproductive biology, and matrix biochemistry, yet these fields differ substantially in experimental models, assay methodologies, isoform definitions, and clinical endpoints. Variability in measuring total osteocalcin (tOCN), the analytically stable N-terminal/mid-region osteocalcin fragment (N-MID), and bioactive ucOCN contribute to inconsistent findings and complicate cross-study interpretation. Pre-analytical factors such as circadian rhythm, vitamin K-dependent γ-carboxylation, and renal function further add to this complexity.

Given this heterogeneity, an integrated and mechanistically grounded synthesis is needed to consolidate OCN-related findings across organ systems. This narrative review aims to unify these perspectives by: (a) summarizing structural determinants and carboxylation-dependent isoform biology; (b) outlining receptor-level mechanisms across target organs; (c) consolidating human evidence, including clamp-validated metabolic data; and (d) clarifying methodological and analytical limitations that critically shape the interpretation of OCN physiology. By integrating insights across disciplines, this review establishes a cohesive physiological framework. It highlights translational implications for metabolic disease, bone disorders, neurocognitive function, male reproductive health, chronic stress, and liver pathology.

This article is a narrative, integrative review that synthesizes current knowledge of OCN across molecular, endocrine, metabolic, neurocognitive, renal, and translational domains. The aim was to combine mechanistic insights with clinical observations, with particular emphasis on bioactive ucOCN and its receptor pathways.

Relevant publications were identified through a non-systematic search of PubMed and Scopus, supplemented by targeted searches in MEDLINE and Web of Science (January 2000–January 2026). Search terms included “osteocalcin”, “undercarboxylated osteocalcin”, “GPRC6A”, “GPR158”, “metabolism”, “NAFLD”, “cognition”, and “vitamin K-dependent proteins”. Additional references were obtained through citation tracking and key review articles. As this is a narrative review, no formal eligibility criteria, protocol registration, or risk-of-bias tools were applied; studies were selected based on relevance, biological coherence, and their conceptual contribution to OCN physiology.

Findings were synthesized qualitatively, accounting for assay heterogeneity involving tOCN, N-MID, and ucOCN, as well as pre-analytical modifiers such as circadian timing, vitamin K-dependent γ-carboxylation, and renal function. Epitope-level assays detecting Glu-OC are not equivalent to full-length ucOCN. These analytical considerations contribute to cross-study variability and frequently complicate the interpretation of OCN-related findings.

Much of the mechanistic knowledge derives from preclinical models of bone–pancreas, bone–liver, and bone–brain signaling, while human evidence remains heterogeneous and highly assay-dependent. Knockout phenotypes differ across mouse strains, and human studies vary in analytical platforms, sampling protocols, and phenotype definitions. These factors underscore the need for standardized ucOCN phenotyping, unified analytical approaches, and context-specific study designs integrating metabolic, endocrine, and renal modifiers.

## 2. OCN Characteristics

### 2.1. Molecular Structure and Isoforms

OCN is a 6 kDa non-collagenous protein composed of 49 amino acids and containing three Gla residues generated through vitamin K-dependent carboxylation [1]. These residues confer high affinity for calcium and hydroxyapatite, enabling OCN’s structural contribution to bone mineralization.

Two major isoforms exist: carboxylated OCN (cOCN)—fully γ-carboxylated, tightly binds hydroxyapatite, and is sequestered in the bone matrix, and ucOCN—lower mineral affinity, circulates systemically, and functions as an endocrine hormone modulating metabolism, male fertility, and cognition [2,3,4].

A concise comparison of both isoforms is provided in Table 1.

### 2.2. Biosynthesis and Carboxylation

**Figure 1:**
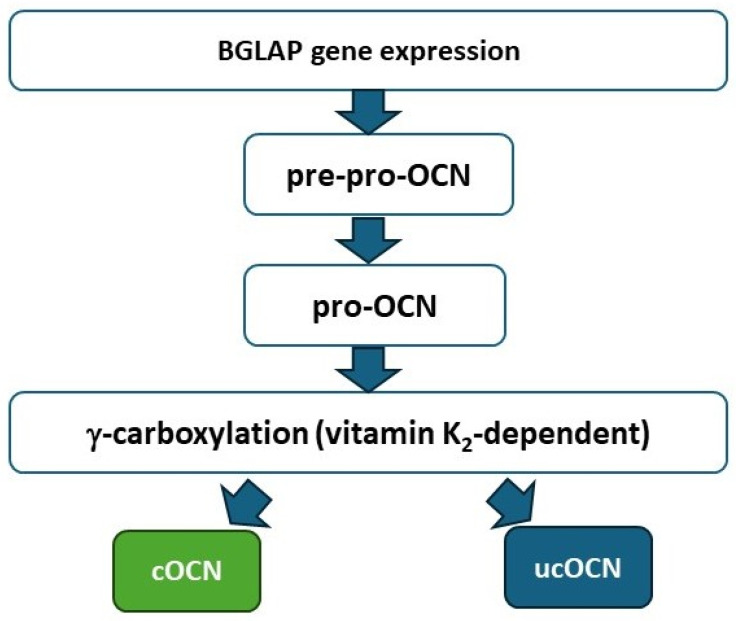
Biosynthesis of Osteocalcin (OCN). OCN is encoded by the bone gamma-carboxyglutamate protein (*BGLAP*) gene and is synthesized as pre-pro-OCN, which is processed to pro-OCN. Vitamin K_2_-dependent γ-carboxylation converts pro-OCN into two major isoforms: cOCN, the fully carboxylated matrix-bound form, and ucOCN, the undercarboxylated, hormonally active circulating form.

OCN synthesis begins with bone gamma-carboxyglutamate protein (BGLAP) transcription (vitamin D-dependent), followed by translation into pre-pro-OCN and proteolytic processing to pro-OCN. Subsequent γ-carboxylation of Glu residues at positions 17, 21, and 24 by γ-glutamyl carboxylase (GGCX), using vitamin K as a cofactor [5,6], generates cOCN; incomplete carboxylation yields ucOCN. Carboxylation increases calcium affinity more than 30-fold [7], promoting stable incorporation of cOCN into the mineral matrix. ucOCN remains largely unbound, circulating as the bioactive endocrine form. Intact OCN is rapidly processed into a stable N-MID, which is the preferred clinical analyte due to its enhanced stability [8]. Figure 1 illustrates the biosynthetic pathway.

### 2.3. Circulating Forms and Clinical Measurement

Serum OCN exists as intact protein and fragments. The N-MID fragment (aa 1–43) offers superior analytical stability [8]. The ucOCN/tOCN ratio reflects vitamin K status and predicts fracture risk, muscle function, and metabolic profiles [9]. Serum OCN levels typically remain below 30 ng/mL and display circadian oscillation, influenced by glucocorticoids [10].

### 2.4. Endocrine Activity

**Figure 2:**
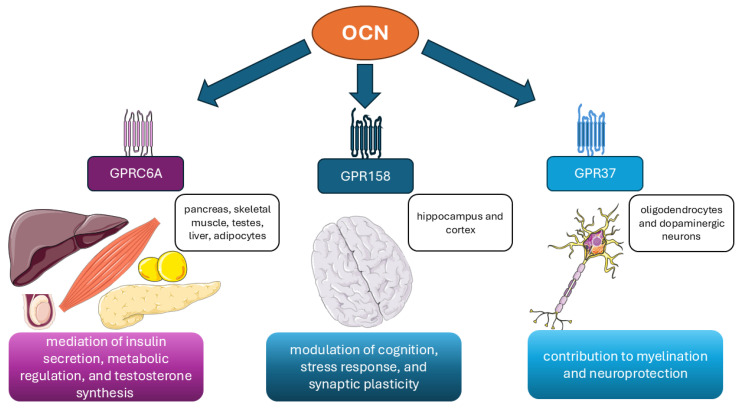
Osteocalcin Signaling Pathways: Tissue-Specific Actions Mediated by GPRC6A, GPR37, and GPR158. Effects of ucOCN action include insulin secretion, improved glucose uptake via protein kinase B (Akt) substrate of 160 kDa (AS160)/glucose transporter type 4 (GLUT4), adiponectin release, hepatic stress reduction (increased nuclear factor erythroid 2-related factor 2 (Nrf2) activity and decreased c-Jun N-terminal kinase (JNK) signaling), Leydig-cell steroidogenesis, and enhancement of hippocampal plasticity through cAMP response element-binding protein (CREB) and brain-derived neurotrophic factor (BDNF). Parts of the figure were created using images provided by Servier Medical Art (https://smart.servier.com), licensed under CC BY 4.0 (https://creativecommons.org/licenses/by/4.0/) (accessed on 20 September 2025).

Bioactive ucOCN signals through its principal receptors—GPRC6A [11], GPR158 [12], and GPR37 [13], mediating metabolic, reproductive, neuromuscular, and neurocognitive effects. These receptors shape tissue-specific responses, including insulin secretion, glucose uptake, adiponectin release, Leydig-cell steroidogenesis, hippocampal plasticity, and stress-related autonomic regulation. A schematic overview of receptor distribution is presented in Figure 2, and detailed receptor pharmacology is discussed in Section 5.2.

### 2.5. Assays and Analytical Considerations

Analytical interpretation of OCN depends strongly on the isoform measured and the assay platform used. In the literature, tOCN, N-MID, and ucOCN are not interchangeable and reflect different physiological signals, and assay specificity varies substantially across platforms [14]. In addition, epitope-level Glu-OC assays may detect OCN fragments and should not be interpreted as equivalent to full-length ucOCN [15].

Pre-analytical modifiers—including circadian timing, sample handling, vitamin K status, and renal clearance—further influence measured concentrations and must be documented in studies using OCN-related markers [16]. For bone-turnover assessment, automated immunoassays targeting the N-MID fragment exhibit superior analytical stability compared with those targeting intact OCN [17]. In contrast, full-length ucOCN—typically quantified using sandwich ELISA—provides the appropriate readout for endocrine and metabolic pathways.

Analytical characteristics and performance differences between the two most commonly used assay systems for ucOCN and Glu-OC measurements (BioLegend ELISA vs. Takara EIA) are summarized in Table 2 [18,19].

To facilitate interpretation across available platforms, Table 2 summarizes key analytical characteristics of the two most used assays for OCN isoforms. The BioLegend sandwich ELISA measures full-length ucOCN and provides higher analytical specificity. In contrast, the Takara Glu-OC EIA detects epitope-level undercarboxylated sites and may cross-react with OCN fragments [18,19]. Differences in target specificity, antibody configuration, detection range, and sample requirements illustrate why tOCN, N-MID, ucOCN, and Glu-OC assays are not interchangeable. These contrasts highlight the importance of assay selection when interpreting OCN-derived markers in both clinical and research settings.

Electrochemiluminescence immunoassay (ECLIA)/N-MID is useful for monitoring antiresorptive therapy; a decrease of at least 20% after 3–6 months indicates treatment response [20]. ucOCN requires interpretation with documented vitamin K status and precise sampling time [21]. In chronic kidney disease–mineral and bone disorder (CKD-MBD), N-MID is preferred for stability, and any OCN measurement should be evaluated together with parathyroid hormone (PTH) and bone-specific alkaline phosphatase (BALP); reduced glomerular filtration rate (GFR) elevates circulating OCN due to impaired clearance, and in dialysis populations, ucOC, percent ucOC, and undercarboxylated matrix Gla protein (dp-ucMGP) may be considered vitamin K-dependent readouts [21].

To provide a consolidated view of the diagnostic and biological roles of OCN-derived markers, a comparative table summarizing the clinical utility of the most discussed OCN-related indices, including the ucOCN/tOCN ratio, the N-MID fragment, and the OC22 peptide, has been presented. This framework highlights the complementary information derived from these markers: the ucOCN/tOCN ratio reflects vitamin K-dependent γ-carboxylation and fracture susceptibility; N-MID serves as a robust, stable indicator of bone turnover in routine clinical practice; and OC22 represents a bioactive domain with emerging translational potential. By presenting these markers side by side, Table 3 clarifies their advantages, limitations, and validation stages in current clinical and preclinical contexts.

To contextualize the clinical interpretation of OCN concentrations, Table 4 summarizes representative serum OCN levels across age groups and endocrine states. These data highlight the physiological variability of OCN and demonstrate how its interpretation depends strongly on growth status, bone turnover activity, and growth hormone (GH)-related endocrine conditions. In pediatric patients with growth disorders, OCN levels are markedly lower and remain poorly standardized, reflecting immature bone metabolism rather than pathological suppression. In contrast, healthy adults exhibit substantially higher concentrations within well-established reference ranges, making OCN a robust marker of bone formation in clinical practice. Adults with growth hormone deficiency (GHD) typically show intermediate values consistent with reduced bone turnover. In contrast, growth hormone (GH) replacement induces a pronounced rise in OCN—often exceeding 60 ng/mL—mirroring the anabolic skeletal response. By presenting these population-specific profiles side-by-side, Table 4 clarifies the physiological dynamic range of OCN and underscores the importance of endocrine context when interpreting the marker.

The table summarizes representative serum OCN values across pediatric and adult populations, highlighting how OCN reflects skeletal maturity, GH and insulin-like growth factor 1 (IGF-1) activity, and overall bone turnover. In children with GH-related growth disorders, OCN is markedly reduced, with values typically below 1.03 ng/mL, supporting its diagnostic utility for distinguishing growth hormone deficiency (GHD) from idiopathic short stature (ISS). Healthy children demonstrate higher, age-dependent concentrations that reflect dynamic skeletal growth. Adults show lower and more stable reference intervals, while individuals with GHD present with suppressed OCN values that increase substantially during GH replacement therapy, reflecting an anabolic skeletal response. These reference intervals and endocrine contexts provide a coherent framework for interpreting OCN across developmental and hormonal states.

## 3. OCN and Bone Metabolism

### 3.1. Role in Bone Turnover and Mineralization

The extracellular matrix of bone tissue comprises collagen fibrils, non-collagenous proteins, and inorganic minerals [28] and is a key determinant of bone properties. Therefore, OCN, as the most abundant non-collagenous protein in bone tissue, has attracted considerable research attention [10,15].

OCN was initially thought to promote bone mineralization due to its Gla residues; however, studies have not demonstrated its presence at the initial sites of mineralization, and it is instead dispersed throughout the bone matrix and concentrated at osteoclast-formed reversal lines [29]. OCN supports bone mineralization by regulating the formation and integration of hydroxyapatite crystals in the bone microenvironment, as confirmed by recent in vitro and in vivo studies [18]. Other researchers have shown that warfarin inhibition of OCN carboxylation in rats leads to abnormal calcification and impaired bone mass growth [30]. Further studies have disclosed that OCN promotes chemotaxis and differentiation of osteoclast precursor cells, suggesting a role in bone resorption [31]. Another study observed that OCN enhances osteoblast function by promoting bone formation and mineralization [32]. Multiscale structural analyses have demonstrated that OCN deficiency leads to smaller mineral crystals, reduced crystallinity and crystal alignment, and altered carbonate substitution, indicating impaired bone material quality independent of bone mass [33]. Together, these findings illustrate the apparent contradictions in the literature and highlight the complexity of OCN’s dual role in bone formation and resorption [34].

OCN also interacts with other non-collagenous matrix proteins, such as osteonectin, osteopontin (OPN), and bone sialoprotein, which play crucial roles in mineralization and resorption [34].

Gene knockout studies have further complicated the issue. OCN-deficient mice from different laboratories have exhibited divergent skeletal phenotypes. The first *Ocn*^−/−^ mice [35] demonstrated increased trabecular bone mass and enhanced osteoblast function. In contrast, another study [36] reported increased trabecular bone mass and osteoblast numbers in female *Ocn*^−/−^ mice on a C57BL/6J background. In contrast, an analysis across several genetic backgrounds found no differences in trabecular or cortical parameters between *Ocn*^−/−^ and wild-type mice [37]. These inconsistencies indicate that both genetic background and experimental design substantially influence the observed skeletal phenotype.

A comprehensive reassessment of *Ocn*^−/−^ models clarified that many of the previously reported discrepancies stemmed from differences in mouse strain, variation in the anatomical region analyzed, and methodological inconsistencies in metabolic testing [38].

Multiscale validation work [39] further demonstrated that OCN and its derived peptide OC22 regulate biomineralization, identifying OC22 as the functionally critical domain responsible for OCN’s bioactivity. In addition, ucOCN has been shown to inhibit chondrocyte hypertrophy and attenuate osteoarthritis development through the GPRC6A/hypoxia-inducible factor 1-alpha (HIF-1α) signaling cascade [32].

### 3.2. Bone Material Quality and Glycation

OCN contributes to bone toughness by forming reversible sacrificial bonds with hydroxyapatite, supporting dissipation of mechanical energy and delaying microcrack propagation [39,40]. This property enhances bone strength beyond what can be inferred from bone mineral density (BMD) measurements alone.

Glycation of bone matrix proteins, including OCN, leads to the accumulation of advanced glycation end-products (AGEs) and advanced glycoxidation end-products (AGOEs). These modifications stiffen collagen, weaken OCN–mineral interactions, and impair nanoscale mechanical behavior of bone [40,41]. As a result, bone fragility increases even when BMD remains within the normal range, a pattern characteristic of aging and diabetes [38,42].

Biochemical markers such as urinary pentosidine provide fracture-risk information independent of BMD and trabecular bone score (TBS), especially in patients with diabetes, where conventional turnover markers may be low [43]. Non-invasive measurements of tissue AGEs, such as skin or lens autofluorescence, correlate with reduced BMD and increased osteoporosis risk even in non-diabetic individuals, confirming that glycation represents a distinct “material quality” phenotype [44].

At the cellular level, AGE adducts such as carboxymethyl-lysine (CML) activate RAGE signaling in osteocytes. This promotes the production of inflammatory cytokines, alters gene expression relevant to bone remodeling, and reduces osteocyte viability [45]. OCN is also susceptible to glycation. Glycation-driven modifications of lysine and arginine residues distort OCN’s conformation, reduce its affinity for hydroxyapatite, and impair both mineral templating and sacrificial bond-mediated energy dissipation [45].

Collectively, AGEs, AGOEs, and glycation-modified OCN disrupt collagen–mineral coupling, interfere with intrafibrillar mineralization, and diminish the energy-dissipation capacity of bone [40,41]. These processes contribute to skeletal fragility that is not captured by densitometry alone and highlight bone material quality as a critical determinant of fracture risk. Evidence from bone-resorption disorders, including osteopetrosis and osteoporosis, demonstrates that osteoclast-driven decarboxylation is essential for generating hormonally active ucOCN, underscoring the mechanistic link between resorption dynamics and osteocalcin bioactivity [46].

### 3.3. Metabolic Syndrome: OCN at the Crossroads of Bone and Energy Metabolism

Recent evidence also indicates that vitamin K2 supplementation modulates bone turnover markers, including OCN and ucOCN, with potential metabolic relevance [47]. The definition of metabolic syndrome (MetS) integrates abdominal obesity, elevated triglycerides (TG), hypertension, hyperglycemia, and reduced high-density lipoprotein cholesterol (HDL-C), as established by the joint statement of major international cardiometabolic societies [48]. Visceral adiposity is a central driver of this cluster, contributing to metabolic dysfunction through inflammatory and endocrine mechanisms [49]. In this context, OCN has emerged as a potential modulator of systemic metabolism. Mechanistic studies demonstrate that it participates in energy regulation via insulin signaling in osteoblasts and reciprocal bone–pancreas interactions [50], enhances adiponectin production and insulin sensitivity in adipocytes [51], and integrates bone turnover with whole-body fuel homeostasis [52,53].

Clinical studies consistently show that lower circulating ucOCN is associated with adverse metabolic features—including increased waist circumference, higher TG, elevated fasting glucose, higher blood pressure, and reduced HDL-C—in community-dwelling older adults [54,55]. In patients with type 2 diabetes (T2DM), higher OCN levels correlate positively with insulin sensitivity and insulin secretion [56]. Additional cohort analyses confirm inverse associations between ucOCN and indices of glucotoxicity and adiposity [54,57], and data from osteometabolic and cardiometabolic populations support the role of osteocalcin as a biomarker of metabolic health [58].

Experimental and translational findings demonstrate that OCN stimulates glucagon-like peptide-1 (GLP-1) secretion and improves glucose utilization, providing a mechanistic link between the gut–bone–pancreas axis and glucose regulation [59]. Interventions targeting inflammation and autonomic regulation, such as galantamine, also improve components of MetS and illustrate the multisystemic integration of OCN-related pathways [60]. Prospective analyses show that higher osteocalcin levels are associated with a reduced risk of diabetes and diabetic kidney disease [61]. Finally, circulating ucOCN has been proposed as an estimator of cardiovascular and diabetes risk in individuals with MetS [62].

These clinically observed associations between low circulating ucOCN and multiple adverse metabolic parameters are systematically summarized in Table 5, which compiles population-based evidence across diverse cohorts and metabolic risk profiles.

The mechanistic pathways underlying the association discussed in this section are discussed in Section 5.2.

### 3.4. Clamp-Validated Human Evidence for ucOCN

In overweight and obese postmenopausal women (n = 132), circulating ucOCN showed consistent associations with glucose metabolism, correlating inversely with fasting glucose and the homeostatic model assessment of insulin resistance (HOMA-IR), and positively with established indices of insulin sensitivity derived from the oral glucose tolerance test (OGTT), including the Matsuda index and the simple insulin-sensitivity index from OGTT (SI(is)OGTT). Importantly, ucOCN also correlated with the glucose infusion rate during the hyperinsulinemic–euglycemic clamp (HEC), further reinforcing its association with insulin sensitivity. Participants in the lowest ucOCN quartile exhibited the weakest glucose clearance during OGTT, indicating clinically relevant metabolic differences.

In individuals with severe obesity (n = 16), ucOCN concentrations were substantially lower in those with T2DM compared with non-diabetic subjects and were related to both glycemic control (fasting glucose, glycated hemoglobin (HbA1c)) and the disposition index (DI; insulin secretion rate × insulin sensitivity, ISR × SI), performing better than tOCN in capturing these metabolic variations.

Together, these findings provide clamp-validated evidence in humans supporting ucOCN as a bioactive signal connecting skeletal function with systemic glucose homeostasis [17].

## 4. OCN in Skeletal Physiology

### 4.1. Vitamin K_2_ and Osteocalcin: Mechanisms and Clinical Relevance

The biological activity of OCN depends on vitamin K_2_-dependent γ-carboxylation, a post-translational modification catalyzed by γ-glutamyl carboxylase (GGCX) that transforms OCN into its carboxylated form. cOCN binds hydroxyapatite efficiently and contributes to optimal bone mineralization. In contrast, insufficient vitamin K_2_ results in accumulation of ucOCN, reflecting impaired carboxylation rather than intrinsic changes in bone turnover [5,15].

The gut microbiome contributes to vitamin K_2_ homeostasis, particularly through the production of menaquinone-7 (MK-7) by taxa such as *Bifidobacterium*, *Bacteroides*, and *Bacillus*. These microbial menaquinones complement dietary intake and form a mechanistic link between intestinal ecology, nutrient supply, and OCN activation [63,64,65,66,67].

**Figure 3:**
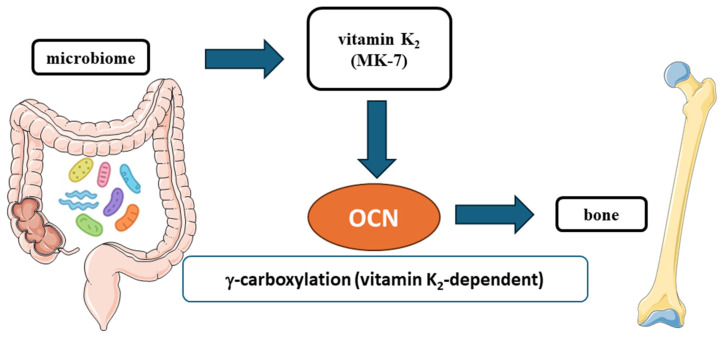
Integrated role of vitamin K_2_, gut microbiome, and OCN carboxylation in bone mineralization. Selected gut taxa synthesize menaquinone-7 (MK-7), which supports γ-carboxylation of OCN via γ-glutamyl carboxylase (GGCX). Carboxylated OCN (cOCN) displays high affinity for hydroxyapatite, enhancing bone mineralization and reducing fracture risk. Parts of the figure were created using images provided by Servier Medical Art (https://smart.servier.com), licensed under CC BY 4. (https://creativecommons.org/licenses/by/4.0/) (accessed on 17 November 2025).

A graphical overview of the microbiome–vitamin K_2_–OCN axis is presented in Figure 3, illustrating MK-7 production, γ-carboxylation of OCN, and the enhancement of bone mineral binding [66,67,68].

**Figure 4:**
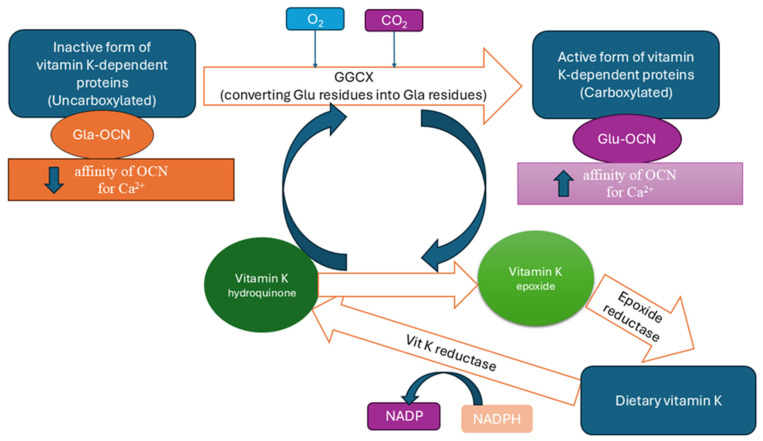
Vitamin K epoxide reductase (VKOR) cycle. The VKOR cycle restores reduced vitamin K, which is necessary for the γ-carboxylation of OCN and other vitamin K-dependent proteins, maintaining continuous bone matrix mineralization.

During γ-carboxylation, vitamin K hydroquinone is oxidized to vitamin K epoxide. The vitamin K epoxide reductase (VKOR) cycle regenerates reduced vitamin K, thereby sustaining the activation of OCN and other vitamin K-dependent proteins [56,64,69,70]. This process is illustrated in Figure 4.

Clinical studies in osteoporosis indicate that vitamin K_2_ status significantly influences OCN γ-carboxylation. Long-term supplementation with MK-7 (180–375 µg/day) consistently reduces ucOCN, improves the ucOCN/cOCN ratio, slows bone mineral density (BMD) loss, and supports structural bone strength [9,71,72,73,74,75,76]. Pharmacologic MK-4 also decreases ucOCN, although its short-term effects on BMD are less consistent [74]. Although these mechanisms originate in skeletal biology, vitamin K_2_-dependent carboxylation becomes even more relevant in chronic kidney disease–mineral and bone disorder (CKD-MBD), where vitamin K metabolism, OCN clearance, and mineral regulation are altered. CKD-specific implications of OCN carboxylation are discussed in Section 5.5.

### 4.2. OCN and Osteoporosis

OCN is closely linked to skeletal remodeling, and its circulating concentrations follow characteristic patterns across physiological and pathological states. In osteoporosis—particularly in postmenopausal women—OCN dynamics reflect the accelerated turnover and changes in bone mineralization that accompany estrogen deficiency. Mechanistic aspects of OCN γ-carboxylation and vitamin K_2_ biology are discussed in Section 4.1; here, the focus is placed on the clinical interpretation of OCN in osteoporotic bone.

Circulating OCN typically increases in high-turnover states such as postmenopausal bone loss, fracture repair, osteomalacia, or tumor-related bone involvement. In contrast, reduced OCN concentrations characterize low-turnover conditions, including hypoparathyroidism, hypothyroidism, chronic glucocorticoid therapy, liver failure, and selected nutritional deficiencies [5,15,47,67,70]. These physiological and pathological settings are summarized in Table 6 and illustrate how OCN trends correspond to the intensity of turnover.

Despite its sensitivity, OCN must be interpreted within the broader biochemical context, as it is influenced by renal clearance, circadian rhythm, and vitamin K status. Therefore, OCN alone does not predict fracture risk; when combined with bone turnover markers such as procollagen type I N-terminal propeptide (PINP) and β-isomerized C-terminal telopeptide of type I collagen (β-CTX), it provides a more reliable representation of skeletal metabolism.

Clinical evidence demonstrates that vitamin K_2_ status modulates OCN γ-carboxylation and influences bone strength. Deficiency increases ucOCN and impairs matrix quality [66,71], while vitamin K_2_—particularly MK-7—lowers ucOCN, improves the ucOCN/cOCN ratio, and supports indices of bone strength. MK-4 reduces ucOCN at pharmacologic doses but has limited short-term effects on BMD. Long-term MK-7 supplementation (180–375 µg/day) produces sustained improvements in OCN carboxylation and bone quality, with site-specific BMD responses dependent on baseline skeletal status [9,73,74,75,76]. These trial data are presented in Table 7.

Altogether, improving OCN carboxylation through adequate vitamin K_2_ intake supports bone quality and may reduce fracture risk, particularly in postmenopausal women. Short-term MK-7 dose–response studies further confirm that even nutritional intakes (10–360 µg/day) significantly enhance γ-carboxylation efficiency and improve the ucOCN/cOCN balance [78]. When interpreted alongside complementary turnover markers, OCN and its carboxylation indices provide a practical, mechanistically grounded tool for assessing skeletal health and guiding nutritional or therapeutic strategies.

## 5. Systemic Endocrine Actions of OCN

### 5.1. Endocrine Physiology of ucOCN

ucOCN acts as a bone-derived hormone linking skeletal remodeling with systemic metabolic, reproductive, cognitive, and stress-related pathways [78,79,80]. It improves glucose homeostasis by stimulating insulin secretion and enhancing insulin sensitivity, as demonstrated in mechanistic, genetic, interventional, and observational studies [78,79,80]. During physical activity, circulating ucOCN levels rise, facilitating more efficient substrate utilization in skeletal muscle and aligning metabolic demand with exercise intensity [79,80]. Beyond glucose regulation, ucOCN supports male reproductive physiology by promoting Leydig-cell maturation and testosterone synthesis [24,81]. Emerging evidence also indicates that ucOCN contributes to the acute stress response [82]. It also crosses the blood–brain barrier and enhances hippocampal plasticity, memory, mood regulation, and emotional resilience [83,84]. In stress physiology, ucOCN increases rapidly and reduces parasympathetic tone, promoting sympathetic activation and rapid metabolic adaptation [85,86]. In hepatocytes, ucOCN reduces endoplasmic-reticulum stress, improves lipid handling, and supports redox homeostasis [84,85,86].

In adipose tissue, ucOCN modulates lipid mobilization through canonical lipolytic enzymes. Adipose triglyceride lipase (ATGL), also known as patatin-like phospholipase domain-containing protein 2 (PNPLA2), is the rate-limiting enzyme initiating lipolysis by catalyzing the first step of triglyceride hydrolysis. ATGL, encoded by the PNPLA2 gene, acts in concert with hormone-sensitive lipase (HSL) to coordinate OCN-linked lipid mobilization in adipocytes. Through GPRC6A-dependent signaling, ucOCN enhances ATGL/HSL-mediated lipolysis, increases adiponectin release, and integrates bone-derived endocrine cues with whole-body energy metabolism.

Taken together, ucOCN functions as a multisystem endocrine messenger integrating bone turnover with whole-body physiology. Key receptor–pathway relationships are summarized in Table 8.

### 5.2. Mechanistic Basis: Osteocalcin Receptors and Signaling (Bone-to-Organ Messaging)

The systemic actions of ucOCN, introduced in Section 5.1, are primarily mediated by two G protein–coupled receptors, GPRC6A and GPR158, with GPR37 playing a supporting role in oligodendrocyte-dependent responses. These receptors differ in their tissue distribution and intracellular coupling, shaping the context-specific effects of ucOCN across metabolic, neurocognitive, reproductive, hepatic, and stress-related pathways [86,87]. Classical knockout studies initially supported endocrine roles of OCN in pancreatic, reproductive, and neuromuscular pathways. Still, more recent models reveal divergent phenotypes and challenge several earlier assumptions, highlighting the need for rigorous assay- and strain-dependent interpretation [88]. These discrepancies have also prompted a critical reevaluation of OCN’s hormonal status, with alternative interpretations suggesting that OCN primarily regulates bone quality rather than acts as a systemic hormone [88,89].

#### 5.2.1. GPRC6A: Central Metabolic and Reproductive Signaling

GPRC6A consists of a large extracellular Venus flytrap (VFT) domain and a seven-transmembrane signaling core. ucCN binds to an allosteric pocket within the VFT cleft, and mutations in key residues such as lysine 352 and histidine 355 markedly reduce ligand efficacy, defining the structural basis of receptor sensitivity [86]. Ligand binding induces closure of the VFT domain. It transmits conformational changes to the seven-transmembrane core, enabling coupling to distinct G-protein subtypes—Gαs, Gαq, or Gαi/o—and thereby supporting tissue-specific signaling [87].

Downstream signaling integrates several canonical pathways relevant to OCN biology, including the phosphoinositide 3-kinase–protein kinase B–mechanistic target of rapamycin pathway (PI3K–Akt–mTOR), the phospholipase C beta–inositol 1,4,5-trisphosphate–calcium pathway (PLCβ–IP_3_–Ca^2+^), the Rat sarcoma GTPase–MAPK/ERK kinase–extracellular signal-regulated kinase cascade (Ras–MEK–ERK), the cyclic adenosine monophosphate–protein kinase A pathway (cAMP–PKA), and AMP-activated protein kinase (AMPK) activation, as summarized in Table 9. These signaling cascades regulate β-cell insulin secretion, muscle glucose uptake, adipocyte thermogenesis, hepatic redox balance, and steroidogenesis, providing the mechanistic foundation for OCN’s multisystem endocrine effects.

#### 5.2.2. GPR158: Neuromodulatory and Synaptic Pathways

GPR158 is the primary neuronal receptor for ucOCN and mediates its effects on learning, memory, and emotional regulation. ucOCN–GPR158 signaling activates CREB/BDNF pathways, enhancing synaptic plasticity, long-term potentiation, and anxiety-related behavioral resilience [12]. In parallel, GPR158 strengthens neurometabolic coupling through insulin receptor substrate (IRS)–PI3K–Akt signaling, supporting astrocytic aerobic glycolysis and the energetic demands of plasticity.

ucOCN also modulates monoaminergic tone by increasing expression of tyrosine hydroxylase and tryptophan hydroxylase-2, boosting dopamine, serotonin, and noradrenaline availability [3]. Together, these pathways position GPR158 as a key mediator linking bone-derived endocrine signals to hippocampal and cortical circuit function. Core mechanisms remain summarized in Table 10.

#### 5.2.3. GPR37 Signaling

ucOCN interacts with GPR37 in oligodendrocytes and dopaminergic pathways, influencing myelin integrity, neuronal protection, and context-dependent motor outcomes [102]. In oligodendrocytes, ucOCN–GPR37 signaling promotes maturation and white-matter stability, while in dopaminergic circuits, it enhances neuronal resilience under metabolic and oxidative stress [102]. Recent evidence further indicates that GPR37 engages broader neuroprotective programs, including ERK/Akt-related kinase signaling and calcium-dependent intracellular processes, which modulate glia–neuron communication, reduce apoptotic susceptibility, and strengthen cellular stress tolerance [103]. Beyond oligodendrocyte development, GPR37 has also been implicated in regulating neuroinflammatory pathways, particularly through IL–6-mediated signaling in oligodendrocyte-driven injury contexts, suggesting a broader role in maintaining neural homeostasis and limiting degenerative processes [104]. Functional consequences of ucOCN–GPR37 signaling and related phenotypes are summarized in Table 11.

#### 5.2.4. Additional Mechanistic Layers of ucOCN Signaling

In intestinal L cells, ucOCN enhances GLP-1 secretion through GPRC6A and a mechanosensitive ion channel (Piezo1), coordinating luminal stretch with Ca^2+^-dependent exocytosis involving synaptosomal-associated protein 25 kDa (SNAP25), synaptotagmin, and syntaxin [105].

ucOCN also contributes to steroidogenesis in Leydig cells via the cAMP–PKA–MEK–ERK–CREB cascade, inducing expression of steroidogenic acute regulatory protein (StAR), cytochrome P450 family 11 subfamily A member 1 (CYP11A1), cytochrome P450 family 17 subfamily A member 1 (CYP17A1), and 3β-hydroxysteroid dehydrogenase (HSD3B). At the same time, testosterone synthesis is further shaped by mitochondrial membrane potential (ΔΨm), nicotinamide adenine dinucleotide phosphate (NADPH) availability, and Ca^2+^-dependent enzymatic activation [106].

At the transcriptional level, ucOCN modulates nutrient- and stress-responsive programs [107], activating forkhead box protein O1 (FOXO1), peroxisome proliferator-activated receptor gamma coactivator-1 alpha (PGC-1α), and peroxisome proliferator-activated receptor alpha/delta (PPARα/δ) to promote mitochondrial biogenesis and fatty-acid oxidation. In parallel, suppression of sterol regulatory element-binding protein 1c (SREBP1c) and carbohydrate-responsive element-binding protein (ChREBP) reduces hepatic lipogenesis, and inhibition of nuclear factor kappa-light-chain-enhancer of activated B cells (NF-κB), together with modulation of activating transcription factor 4 (ATF4), supports anti-inflammatory and proteostatic adaptation.

Epigenetically, ucOCN influences histone acetylation via CREB-associated coactivators, modulates DNA methylation under metabolic stress, and decreases GPR158 promoter methylation with aging, increasing receptor availability [108].

Finally, ucOCN participates in autonomic stress physiology: circulating ucOCN rises within minutes during acute stress, inhibiting acetylcholine synthesis in parasympathetic neurons, disinhibiting sympathetic output, and supporting an adrenal-independent acute stress response [109].

### 5.3. Systemic Actions of OCN

The endocrine actions of ucOCN span multiple organ systems, coordinating glucose metabolism, muscular energy metabolism, gut–pancreas signaling, reproductive function, neurocognitive processes, and acute stress adaptation. These effects arise from the receptor mechanisms detailed in Section 5.2 and summarized in Table 8, which provides an organ- and receptor-centric overview of ucOCN’s actions.

#### 5.3.1. Glucose Homeostasis and Adiposity

ucOCN enhances insulin secretion and sensitivity via GPRC6A-dependent pathways in β-cells and peripheral tissues [24,86,87]. Observational and interventional studies show inverse associations of ucOCN with fasting glucose, HbA1c, adiposity indices, and HOMA-IR [86,87,88,89,90], while variability across trials reflects assay choice and physiological state [74,93,94,95,96,97,98]. Within the broader adipokine network, factors such as omentin-1 also track insulin sensitivity and body fat distribution, shaping the metabolic landscape in which ucOCN exerts its endocrine actions [110]. Key metabolic endpoints across experimental and human studies are summarized in Table 12.

To further dissect OCN-dependent metabolic regulation, multiple genetic models have been developed. *Ocn^−^*/^−^ mice exhibit impaired insulin secretion and glucose intolerance, whereas *Esp*^−/−^ mice display enhanced insulin sensitivity consistent with increased ucOCN bioavailability [2,50,93]. More recent CRISPR-generated *Bglap*/*Bglap2* dKO models show preserved metabolic function, indicating strain-specific compensation [34,96,109].

These complementary genetic findings are systematically compared in Table 13.

While Table 13 summarizes the metabolic phenotypes of *Ocn*^−/−^, *Esp*^−/−^, and *Bglap*/*Bglap2* knockout models, Table 14 integrates evidence across the translational continuum—from receptor-level mechanisms to human interventions and population cohorts. ucOCN promotes insulin secretion and β-cell proliferation through GPRC6A-mediated PI3K/AKT/mechanistic target of rapamycin (mTOR) signaling [11,86], and stimulates adiponectin release and thermogenic programming in adipocytes via Rap1–ERK/CREB activation [24,90]. These pathways establish the mechanistic foundation for bone-derived regulation of glucose and lipid metabolism. Observational meta-analyses extend these insights to human physiology, reporting inverse associations between ucOCN and fasting glucose, BMI, and body-fat percentage [93,94], although causality remains uncertain. Interventional data provide additional nuance: high-dose MK-7 supplementation (375 µg/day for 12 months) substantially reduces ucOCN and raises adiponectin without improving insulin resistance in healthy women [96], whereas combined vitamin D_3_ and K_2_ therapy improves glycemia and HOMA-IR in T2DM patients while altering the ucOCN/cOCN ratio [74]. Physiological studies further demonstrate that ucOCN cooperates with insulin in skeletal muscle to enhance GLUT4 translocation via AS160 phosphorylation, supporting exercise-induced glucose uptake [91,92]. Finally, Mendelian randomization analyses show that higher genetically determined bone mineral density associates with greater T2DM risk and elevated 2 h OGTT glucose [97,98], underscoring the complexity of bone–glucose crosstalk beyond osteocalcin alone. Collectively, these findings reveal mechanistic consistency but clinical heterogeneity, emphasizing the need for standardized ucOCN phenotyping and context-specific study designs. Key mechanistic and translational tiers are synthesized in Table 14.

These findings must be interpreted in light of several methodological considerations. Classical knockout models (*Ocn*^−/−^, *Esp*^−/−^) support impaired insulin secretion, reduced β-cell mass, and altered glucose tolerance, whereas modern CRISPR-generated *Bglap*/*Bglap2* dKO models frequently show no endocrine abnormalities—pointing to strain-dependent compensation and analytical heterogeneity across studies [93,94,95]. These discrepancies indicate that (a) genetic background, (b) assay choice (tOCN/N-MID vs. ucOCN), and (c) pre-analytical handling critically shape phenotypes; thus, mechanistic interpretation requires standardized ucOCN readouts, explicit vitamin K control, and harmonized metabolic endpoints [8,14,17]. For orientation, Table 13 contrasts model phenotypes, while Table 14 positions these findings along the mechanistic–translational continuum.

While ucOCN links bone turnover to systemic energy metabolism, the marked heterogeneity in human data and the strong influence of vitamin K-dependent carboxylation underscore the need for standardized ucOCN/tOCN phenotyping and targeted RCTs focused on glycemic outcomes. In parallel, ucOCN actions extend beyond glucose and adiposity to the gut (GLP-1), brain (GPR158), and testes (GPRC6A), as discussed in the dedicated organ-axis sections.

#### 5.3.2. Gut–Pancreas Incretin Axis: ucOCN and GLP-1 Secretion

ucOCN directly modulates the gut–pancreas axis by stimulating GLP-1 secretion from enteroendocrine L-cells. This effect is mediated through GPRC6A and Piezo1-dependent mechanosensation, which links luminal stretch to Ca^2+^-driven exocytosis of incretin granules [106,107,108]. Through this mechanism, ucOCN amplifies nutrient-stimulated insulin secretion and enhances the postprandial insulinotropic response, positioning bone-derived signaling within the classical incretin framework.

Preclinical studies support a feed-forward loop in which ucOCN increases GLP-1 release, and GLP-1 in turn promotes β-cell proliferation, survival, and insulinotropic sensitivity. Although human evidence remains limited, available data indicate physiological plausibility of a bone–gut–pancreas circuit that integrates skeletal remodeling with glycemic control [106,107,108].

Mechanistic elements of this axis, including GPRC6A, Piezo1, Ca^2+^ influx, and downstream exocytotic machinery (SNAP25, synaptotagmin, syntaxin), are summarized in Table 15.

#### 5.3.3. Bone–Muscle Axis: Skeletal Muscle and Exercise Adaptation

The bone–muscle endocrine loop is one of the best-characterized systemic actions of ucOCN. During endurance exercise, circulating ucOCN rises rapidly and acts on GPRC6A in myofibers to enhance AS160-dependent GLUT4 translocation, increasing glucose uptake and improving mitochondrial coupling [4]. ucOCN further synergizes with contraction-induced IL-6, forming a feed-forward muscle–bone–muscle loop that optimizes endurance performance [109,111]. Recent multiscale analyses confirm that ucOCN-dependent AS160 phosphorylation underlies these adaptations and refine the interpretation of bone–muscle crosstalk in modern *Ocn*^−/−^ models [112].

A structured overview of the key mechanistic nodes—including GPRC6A engagement, AS160/GLUT4 signaling, contraction synergy, and IL-6-mediated amplification—is presented in Table 16.

**Figure 5:**
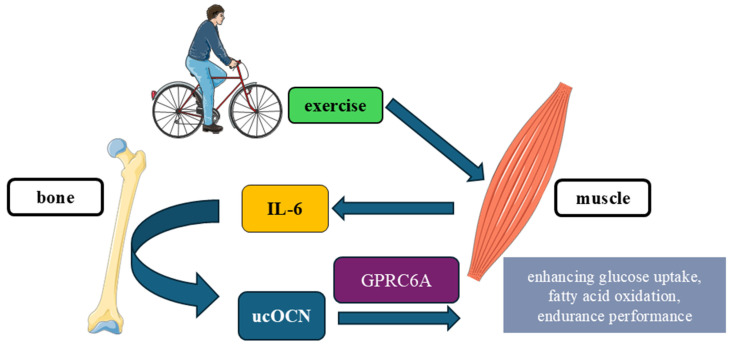
Bone–muscle axis during exercise. Exercise increases muscle-derived interleukin 6 (IL-6), which signals to bone and enhances the activation of undercarboxylated osteocalcin (ucOCN); ucOCN then acts on skeletal muscle via G protein-coupled receptor class C group 6 member A (GPRC6A), supporting glucose uptake, fatty acid oxidation, and endurance performance. Parts of the figure were created using images provided by Servier Medical Art (https://smart.servier.com), licensed under CC BY 4. (https://creativecommons.org/licenses/by/4.0/) (accessed on 15 December 2025).

The muscle–bone–muscle loop engaged during exercise is depicted in Figure 5, showing IL-6 signaling from contracting muscle to bone, the activation of ucOCN, and its GPRC6A-dependent action in skeletal muscle.

In summary, ucOCN/GPRC6A signaling primes working muscle to take up glucose efficiently and utilize fatty acids during endurance, while the IL-6–bone–ucOCN loop amplifies this response. Human translation appears state-dependent (training status, insulin sensitivity, vitamin-K carboxylation) and assay-dependent (ucOCN vs. tOCN), and these variables should be controlled in future trials.

#### 5.3.4. Bone–Testis Axis: Leydig Cell Steroidogenesis via GPRC6A

ucOCN acts directly on Leydig cells via GPRC6A, triggering the canonical cAMP–PKA–MEK–ERK–CREB signaling cascade that induces key steroidogenic enzymes—including StAR, CYP11A1, CYP17A1, and HSD3B—and thereby promoting testosterone synthesis partly independently of the classical hypothalamic–pituitary–gonadal axis [1,113,114,115,116]. This mechanism establishes a bone–testis endocrine link.

Genetic models strongly support this framework: *Ocn^−^*/*^−^* mice exhibit reduced Leydig-cell maturation, impaired spermatogenesis, and low testosterone despite elevated luteinizing hormone (LH), whereas *Esp*^−^**/**^−^ mice—characterized by increased ucOCN bioavailability—display enhanced reproductive metrics [113]. Leydig-specific *Gprc6a* conditional knockout mice phenocopy the *Ocn^−^*/*^−^* phenotype, confirming the requirement of GPRC6A for ucOCN action [113]. Cell-based studies in porcine and buffalo Leydig cells further corroborate this mechanism: pharmacological or genetic blockade of the GPRC6A-dependent signaling cascade suppresses steroidogenesis [117,118]. Key reproductive phenotypes from these genetic models are summarized in Table 17.

In Klinefelter syndrome, tOCN reflects HPG-driven bone turnover and declines after testosterone therapy; therefore, it should not be interpreted as a surrogate of ucOCN bioactivity in the Leydig pathway. For evaluation of the bone–testis axis, ucOCN (with vitamin-K phenotyping) is the appropriate endocrine readout, whereas total OCN primarily reflects bone remodeling.

Evidence from human studies supports this framework: circulating ucOCN shows a positive association with testosterone, and several GPRC6A variants (including rs2247911) are associated with testicular endocrine traits [115,116,119,120]. Together, these findings support ucOCN–GPRC6A as a conserved axis in male reproductive physiology, while also highlighting the need for standardized ucOCN phenotyping and vitamin-K–aware study designs.

From a translational perspective, phenotyping panels combining ucOCN, LH, testosterone, and relevant GPRC6A variants may refine the assessment of metabolic hypogonadism. Interventions that increase ucOCN—such as improved insulin sensitivity or exercise training—may enhance testicular steroidogenesis, while GPRC6A-positive allosteric modulators offer a potential therapeutic avenue [50,81].

**Figure 6:**
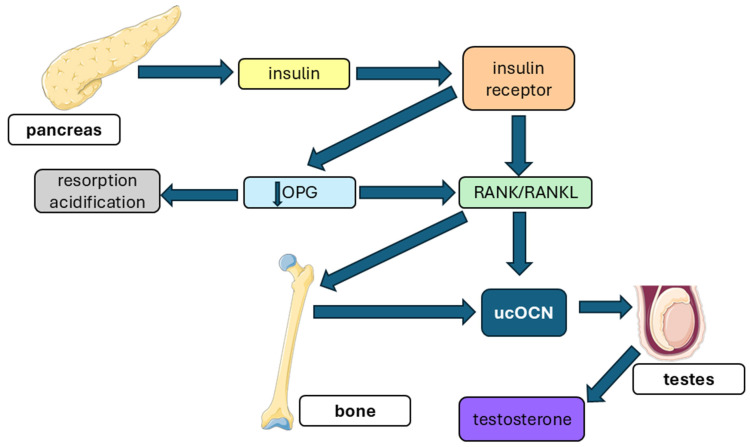
Insulin–bone–testis endocrine axis mediated by RANK/RANKL signaling and ucOCN. Insulin released from the pancreas binds to insulin receptors on osteoblasts, stimulating receptor activator of nuclear factor κB ligand (RANKL) expression and enhancing osteoclast activation. Increased bone resorption and local acidification promote the release of ucOCN into the circulation. Circulating ucOCN then acts on the testes to enhance testosterone synthesis. Osteoprotegerin (OPG) serves as a decoy receptor for RANKL, inhibiting osteoclast differentiation and reducing bone resorption. Parts of this figure were created using images provided by Servier Medical Art (https://smart.servier.com), licensed under CC BY 4.0 (https://creativecommons.org/licenses/by/4.0/) (accessed on 17 January 2026).

Mechanistic integration (bone–pancreas–testis loop): insulin signaling in osteoblasts reduces OPG and enhances RANKL-mediated osteoclast activation, generating the acidic microenvironment required to decarboxylate OCN to its bioactive form, ucOCN. The resulting rise in circulating ucOCN engages the allosteric VFT domain of GPRC6A on Leydig cells, activating downstream steroidogenic signaling and ultimately promoting testosterone synthesis [50,81,116]. This integrated pancreas–bone–testis circuit is illustrated in Figure 6.

Clinical implications: phenotyping strategies combining ucOCN, LH, testosterone, and selected GPRC6A variants may improve evaluation of metabolic hypogonadism. Interventions that increase ucOCN (exercise, insulin-sensitizing strategies) may augment testicular output, and pharmacologic GPRC6A-positive allosteric modulators offer a potential future therapy [50,81].

In contrast to the well-defined bone–testis axis, a corresponding ovarian pathway remains largely speculative. Current human and preclinical evidence provides no definitive proof of ucOCN-mediated ovarian signaling, and available findings are limited, heterogeneous, and often indirect [81,88]. A comparative reading of the male pathway underscores this asymmetry: whereas GPRC6A-dependent steroidogenic mechanisms in Leydig cells are well established, receptor-level validation, ovarian tissue phenotyping, and controlled human studies are lacking for any putative ovarian arm. Given this evidence gap, mechanistic assertions regarding OCN-driven ovarian regulation remain premature, and future work will require receptor confirmation, cell-specific models, and vitamin K-aware phenotyping.

Postnatally, osteocalcin remains available to the developing infant through breast milk, where its concentration declines physiologically across lactation [121]. Although the functional relevance of milk-borne OCN for human neurodevelopment remains uncertain, this perinatal continuity aligns conceptually with the prenatal ucOCN–GPR158 axis and provides a developmental bridge to later cognitive effects discussed below.

#### 5.3.5. Neurogenesis and Cognition—OCN as a Neuromodulator

ucOCN functions as a neuromodulator that integrates synaptic plasticity, neurochemical signaling, neurometabolic support, and stress-related adaptation within the central nervous system. After crossing the blood–brain barrier, ucOCN binds GPR158 on hippocampal pyramidal neurons, engaging two convergent programs central to cognition [12].

First, ucOCN activates CREB–BDNF signaling, strengthening long-term potentiation (LTP), paired-pulse facilitation (PPF), and memory consolidation, while reducing anxiety-like behavior.

Second, ucOCN links synaptic plasticity to energy supply through IRS–PI3K–Akt-dependent support of astrocytic aerobic glycolysis, providing the metabolic budget for activity-dependent synaptic remodeling [12].

Beyond glutamatergic mechanisms, ucOCN reshapes the neurochemical milieu by increasing tryptophan hydroxylase-2 and tyrosine hydroxylase expression, elevating serotonin, dopamine, and noradrenaline. Simultaneously, ucOCN attenuates GABAergic tone via downregulation of *Gad1*/*Gad2*—a signature consistent with antidepressant-like phenotypes in preclinical models [3]. Core mechanisms across the bone–brain axis are summarized in Table 18.

During gestation, maternal ucOCN crosses the placenta, preventing neuronal apoptosis and preserving hippocampal architecture; loss of maternal osteocalcin leads to ventriculomegaly and lifelong deficits in hippocampus-dependent learning [3]. This prenatal influence establishes the trajectory for adult plasticity and is coherent with the ucOCN–GPR158 axis [12].

In adulthood and ageing, osteocalcin restores hippocampal plasticity and normalizes synaptic throughput; GPR158 is required for these cognitive effects [12]. Human Mendelian randomization analyses suggest a protective, metabolism-linked association between higher osteocalcin levels and lower Alzheimer’s disease risk. Across disease models, a common mechanistic core emerges: osteocalcin enhances BDNF signaling and induces hippocampal autophagy in Alzheimer’s disease [122], engages GPR37-dependent myelin and dopamine pathways in Parkinson’s disease [13], and diverts glucose flux toward the pentose phosphate pathway (PPP) after ischemic stroke, reducing pyroptosis and supporting neuronal survival [123]. Together, these findings highlight OCN’s role in aligning plasticity, metabolic resilience, and neuroimmune restraint across diverse conditions.

Beyond hippocampal cognition, GPR158 modulates local circuit excitability by reducing M-current thresholds, enhancing presynaptic release efficiency, and supporting both short- and long-term plasticity [12]. Reviews emphasize its circuit- and state-dependent function, consistent with variable regional effects [124]. Emerging evidence includes OCN-responsive network-level activity shifts in regions enriched for GPR37/GPR158 expression, demonstrated using pharmacological manipulations and resting-state functional MRI (fMRI) [124]. In addition, ucOCN suppresses the unfolded protein response (UPR)—including Heat Shock Protein Family A Member 5 (HSPA5), X-box Binding Protein 1 (XBP1), and C/EBP Homologous Protein (CHOP)—under endoplasmic reticulum (ER) stress [125], and hyperglycemia-induced epigenetic repression of GPR158 may further weaken bone–brain communication [126]. These developments are summarized in Table 19.

#### 5.3.6. Acute Stress Response—ucOCN as a Rapid Endocrine Signal

Beyond its metabolic and neurocognitive functions, ucOCN acts as a rapid-acting endocrine mediator during the acute stress response (ASR). In mice, rats, and humans, circulating bioactive osteocalcin increases within minutes of diverse stressors and contributes to the “fight-or-flight” phenotype by reducing parasympathetic tone and unmasking sympathetic output, thereby facilitating increases in heart rate, ventilation, and glucose availability independently of adrenal catecholamines [127]. Mechanistically, acute stress triggers glutamate uptake in osteoblasts, preventing osteocalcin inactivation and enabling its immediate release into the circulation. Circulating OCN then acts on postganglionic parasympathetic neurons, suppressing acetylcholine synthesis and shifting the autonomic balance toward sympathetic dominance [127,128].

OCN also interfaces with adrenal physiology and the HPA axis. Embryonic OCN is required for normal adrenal development and glucocorticoid/mineralocorticoid biosynthesis, and loss of signaling reduces steroidogenic differentiation and blunts the corticosterone rise during acute stress [129]. Parallel immunomodulatory actions—such as attenuation of nuclear factor kappa B (NF-κB) signaling and context-dependent anti-inflammatory effects—further support tissue resilience under stress [82].

Recent findings further demonstrate that embryonic osteocalcin signaling is required for normal adrenal development and lifelong steroidogenic capacity, providing a mechanistic basis for its rapid endocrine involvement during acute stress responses [130]. Human stress paradigms, especially the Trier Social Stress Test (TSST), provide a standardized framework for integrating autonomic, endocrine, and affective responses. TSST produces reproducible increases in cortisol, heart rate, blood pressure, and anxiety, with timing and amplitude that correspond to minute-scale ucOCN surges [131,132,133]. These human data strengthen the translational relevance of the OCN–ASR axis observed in rodent and primate models. Additional evidence demonstrates adrenal-independent ASR preservation: both adrenalectomized rodents and patients with adrenal insufficiency display intact stress responses accompanied by elevated circulating OCN, indicating that OCN provides a parallel, adrenal-independent route of acute stress signaling [127,128]. Neuro–skeletal communication also contributes to the ASR. Manipulations of the basolateral amygdala (BLA) alter osteoblast-derived OCN release and reshape stress responses, demonstrating a brain → bone glutamatergic pathway that modulates rapid endocrine responses [127,128].

Exercise, a physiological stressor, similarly elevates total and bioactive OCN; muscle-derived IL-6 signals to osteoblasts to promote OCN release, while OCN feeds back to improve muscular fuel utilization [134,135].

Collectively, these strands establish osteocalcin as a multisystem stress hormone integrating autonomic, adrenal, metabolic, and neuroimmune components. Its biomarkers and pathways hold translational potential for stress-exacerbated metabolic and neuropsychiatric conditions, as summarized in Table 20 [136,137].

References [127,128] provide the core experimental foundation for most acute stress response findings summarized in this table.

### 5.4. OCN at the Liver–Gut Interface: Mechanistic Protection from Non Alcoholic Fatty Liver Disease (NAFLD)

ucOCN exerts hepatoprotective effects through coordinated antioxidant, lipogenic-repressive, and enterohepatic mechanisms. In hepatocytes, ucOCN activates Nrf2 and suppresses JNK, thereby mitigating oxidative and endoplasmic-reticulum stress and reducing steatosis in diet-induced NAFLD [138]. Genetic models confirm causality: hepatocyte-specific deletion of Gprc6a abolishes ucOCN-mediated protection, establishing a hepatic OCN → GPRC6A axis that limits lipogenesis and supports lipid clearance [139].

A translational extension is provided by chemically synthesized osteocalcin (csOCN), which activates AMPK → FOXO1/BCL6 signaling, suppresses CD36, and reduces hepatic fatty-acid uptake in NAFLD models [140]. Docking and colocalization studies suggest a direct csOCN–CD36 interaction with higher affinity than oleate or palmitate, nominating CD36 as a tractable therapeutic node [140].

Native ucOCN also stimulates AMPK, which downregulates SCD1, attenuating de novo lipogenesis [141]. Together, ucOCN’s Nrf2/JNK buffering, AMPK/SCD1 repression, and csOCN’s CD36 targeting converge on reduced hepatic lipid influx and synthesis [138,139,140,141]. These findings remain preclinical; human-level pharmacology and dose-finding studies are required. Although csOCN–CD36 interference demonstrates robust anti-steatotic activity in preclinical models, these findings remain predominantly rodent-focused. Translation to humans will require a first-in-human safety evaluation, pharmacokinetic and stability profiling, and receptor-level validation to clarify tissue selectivity and dose scalability. While the therapeutic potential of csOCN is promising, it remains experimental until supported by controlled early-phase clinical trials.

Human data align directionally with preclinical models. In biopsy-characterized and prospective cohorts, lower circulating OCN associates with more severe steatosis/fibrosis and—with sex-specific patterns—a higher NAFLD incidence and lower remission [142].

Part of OCN’s hepatic benefit likely depends on the enterohepatic incretin axis. ucOCN stimulates GLP-1 secretion and requires GLP-1R signaling for full metabolic benefit [143,144]. Mechanosensitive Piezo1 further strengthens GLP-1 output [145], and the central hepatoprotective role of Nrf2 across liver injury models underscores the relevance of the ucOCN—Nrf2 axis [146].

A consolidated overview of hepatic signaling, csOCN mechanisms, incretin crosstalk, and human evidence is provided in Table 21.

### 5.5. OCN and Chronic Kidney Disease–Mineral and Bone Disorder (CKD-MBD)

Progressive loss of kidney function leads to the constellation of abnormalities collectively termed CKD-MBD, encompassing dysregulated calcium–phosphate homeostasis, secondary hyperparathyroidism, impaired skeletal remodeling (renal osteodystrophy), and extra-osseous calcification [147,148,149,150]. Contemporary Kidney Disease: Improving Global Outcomes (KDIGO) guidance emphasizes integrating biochemical parameters (Ca, P, PTH, 25(OH)D) with bone phenotype and fracture risk, while recognizing that many BTMs become unreliable in advanced CKD.

Consensus recommendations from the International Osteoporosis Foundation (IOF), the International Federation of Clinical Chemistry and Laboratory Medicine (IFCC), and the European Society for Clinical and Economic Aspects of Osteoporosis, Osteoarthritis and Musculoskeletal Diseases (ESCEO) highlight bone-specific alkaline phosphatase (BALP), intact procollagen type I N-terminal propeptide (intact PINP), and tartrate-resistant acid phosphatase isoform 5b (TRACP-5b) as preferred BTMs in CKD because they are minimally influenced by renal clearance, whereas beta–C-terminal telopeptide of type I collagen (β-CTX-I) and several OCN formats accumulate with declining GFR [5,14,151].

In CKD, circulating OCN concentrations may rise due to reduced renal clearance of intact and fragmentary forms. N-terminal/mid-region osteocalcin fragment (N-MID) is preferred for analytical and pre-analytical stability. OCN should be interpreted only together with PTH and BALP, and, where available, intact PINP and TRACP-5b. Sampling time and vitamin K status should be documented, as functional vitamin K deficiency—indicated by elevated percentages of ucOCN (%ucOC) and dp-ucMGP—is frequent in dialysis cohorts and substantially alters OCN carboxylation patterns [8,14,152,153].

#### 5.5.1. Functional Vitamin K Deficiency in Dialysis: OCN Readout

Functional vitamin K deficiency is highly prevalent in patients undergoing hemodialysis and typically manifests as elevated percentages of undercarboxylated osteocalcin (%ucOC) and dp-ucMGP. Short-term supplementation with MK-7 has been shown to correct these biochemical abnormalities more effectively than a vitamin-K-rich diet, as demonstrated in a six-week randomized crossover study in which MK-7 produced larger reductions in ucOC and dp-ucMGP. Longer-term skeletal effects, however, appear site-specific: in the two-year RenaKvit randomized controlled trial, MK-7 supplementation helped preserve lumbar-spine bone mineral density while accelerating bone loss at the one-third distal radius. A meta-analysis of dialysis cohorts confirms consistent biochemical improvements—most prominently reductions in ucOC and dp-ucMGP—without corresponding evidence of regression in vascular calcification [153,154,155,156].

Taken together, these findings support targeted biochemical correction of vitamin K deficiency in CKD-MBD, while underscoring the need for caution when extrapolating improvements in vitamin-K-dependent biomarkers to clinical outcomes such as fracture reduction or slowed calcification progression.

#### 5.5.2. Pediatric CKD and Vitamin-K–Dependent Biomarkers

In pediatric CKD, including children receiving hemodialysis, circulating ucOCN is consistently elevated compared with healthy peers, and this increase has been shown to predict fracture risk independently. BALP typically rises in parallel, supporting its value as a companion marker of turnover in the pediatric CKD–MBD setting. Together, these findings position ucOCN as a promising fracture-risk biomarker in children with CKD, although validation in larger, longitudinal cohorts remains necessary [157]. Beyond skeletal outcomes, expanding the assessment to include vascular indicators may refine clinical risk stratification; for example, combining bone-turnover markers with surrogate measures such as carotid intima–media thickness (CIMT) has been proposed to capture integrated skeletal–vascular risk across CKD stages [158].

To contextualize these pediatric observations, Table 22 summarizes CKD-appropriate bone turnover and vitamin K-dependent biomarkers for both adult and pediatric CKD. It outlines renal dependence, analytical considerations, and clinical utility for markers such as PTH and BALP—central KDIGO/KDOQI anchors in CKD–MBD [147,148]—as well as intact PINP and TRACP-5b, which retain interpretive value due to minimal renal retention [14,151]. In contrast, β-CTX-I becomes unreliable beyond CKD G3 due to its dependence on renal clearance [14,151].

OCN (total or N-MID) remains useful when interpreted alongside PTH and BALP, with elevations reflecting both impaired clearance and true turnover changes [5,14,151]. N-MID offers improved pre-analytical stability but still requires contextual interpretation with primary turnover markers [8,14]. Vitamin-K-dependent biomarkers—including ucOC, %ucOC, and dp-ucMGP—inform carboxylation status, respond sensitively to MK-7 supplementation, and are best measured at baseline and 6–12 weeks after dietary or supplementation interventions [152,153,154,157]. dp-ucMGP additionally reflects vascular calcification-related pathways, although assay variability and endpoint uncertainty persist [153,156].

Table 22 provides a consolidated reference for applying these biomarkers in clinical practice across CKD stages G3–G5D, supporting the integration of bone and vitamin-K-dependent markers in both pediatric and adult populations.

#### 5.5.3. Interpretation of OCN in CKD–MBD

Interpreting OCN in CKD–MBD requires a structured, clinically anchored approach in which OCN remains a supportive rather than decisive biomarker. The assessment begins with correct turnover phenotyping, ideally using PTH and BALP as first-line indicators of skeletal remodeling, and expanding the panel to include intact PINP and TRACP-5b when available. These markers retain interpretability across CKD stages and help distinguish whether biochemical changes are driven by altered turnover or reduced renal clearance [147,151]. Within this framework, OCN—preferably measured as the N-MID fragment—should be interpreted only together with PTH and BALP, since elevations can arise from both impaired clearance and changes in bone turnover. Consequently, OCN should not drive therapeutic decisions independently in CKD stages G4: estimated glomerular filtration rate (eGFR) 15–29 mL/min/1.73 m^2^–G5D: stage 5 on dialysis [14,151].

A second interpretive dimension concerns the vitamin K axis, which significantly affects OCN carboxylation. In dialysis patients or whenever vitamin K deficiency is suspected, assessment of ucOC, %ucOC, and dp-ucMGP provides insight into γ-carboxylation status. When these markers are elevated, dietary optimization or supplementation with MK-7 is recommended, followed by biochemical reassessment after approximately 6–12 weeks to evaluate improvement [153,154,155,156,157]. In this context, changes in OCN may signal modifications in vitamin K status rather than shifts in bone turnover.

Pediatric CKD requires special consideration. In children, ucOCN together with BALP has been proposed as a fracture-risk indicator, complementing clinical and imaging data. Several pediatric cohorts have demonstrated that higher ucOCN can independently predict skeletal fragility in CKD, although this application remains under refinement [157].

Monitoring should integrate CKD stage, turnover phenotype, and therapy. PTH, BALP, intact PINP, and TRACP-5b are typically reassessed every 3–6 months, depending on treatment intensity. β-CTX-I should be reserved for CKD stages ≤ G3 (eGFR 30–59 mL/min/1.73 m^2^) because of renal retention at more advanced stages. OCN is most useful when interpreted as supportive evidence rather than as a stand-alone determinant of turnover or treatment response [5,14,147,151]. This tool becomes especially informative when contextualized with vitamin K-related markers in dialysis patients or with growth-related bone physiology in children.

The broader evidence informing this interpretative model spans several domains. KDIGO and KDOQI guidelines outline stage-dependent biochemical monitoring (Ca, P, PTH, 25(OH)D) and endorse PTH and BALP as anchors of turnover assessment [147,148]. ESCEO recommendations specify BALP, intact PINP, and TRACP-5b as preferred BTMs in adults and caution against β-CTX-I in advanced CKD due to its renal retention [5,14,151]. N-MID OCN is characterized by superior pre-analytical stability, although total and fragmentary OCN increase with reduced clearance; therefore, OCN values require paired interpretation with PTH and BALP to avoid mis-driven decisions [8,14]. Vitamin K evidence includes randomized crossover studies demonstrating that MK-7 lowers ucOC and dp-ucMGP more effectively than dietary interventions, as well as the RenaKvit randomized controlled trial, which reported site-specific BMD responses. Several meta-analyses have confirmed biochemical improvement with generally neutral structural outcomes in CKD populations [153,154,155,156]. Pediatric studies indicate that ucOC may help identify children at risk of fracture, whereas BALP tracks turnover across growth stages [157]. Finally, in adults, combining BTMs with vascular assessments such as carotid intima–media thickness may refine integrated skeletal–vascular risk stratification in CKD [158].

## 6. OCN and Atherosclerosis: Mediator or Marker of Vascular Risk

### 6.1. Mediator vs. Marker: Bench, Artery, and Human Context

OCN sits at the crossroads of skeletal and vascular physiology, where two biological identities diverge. Experimental data consistently show that physiological ucOCN is vascularly neutral or mildly favorable: it does not impair vasorelaxation in rabbit aorta nor worsen endothelial indices in human cell systems, and it aligns with nitric oxide-supportive signaling and reduced stress- response activity in vascular cells [159,160]. Yet in vivo these effects frequently mirror systemic metabolic improvements rather than artery-autonomous ucOCN action, underscoring the need to distinguish endocrine from vascular-compartment signals [159].

**Figure 7:**
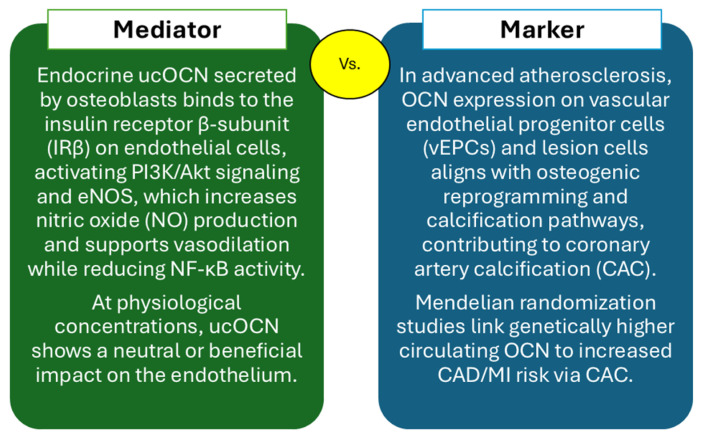
Osteocalcin as an endothelial mediator (left panel) and a vascular calcification marker (**right panel**). (**Left panel**): Undercarboxylated osteocalcin (ucOCN) engages insulin receptor β-subunit (IRβ) and downstream PI3K/Akt–eNOS pathways, supporting nitric oxide (NO) production and reducing NF-κB signaling. Right panel: Osteocalcin (OCN) expression in vascular endothelial progenitor cells (vEPCs) reflects osteogenic reprogramming and contributes to coronary artery calcification (CAC), a process linked to higher risk of coronary artery disease (CAD) and myocardial infarction (MI).

This dual perspective becomes clearer when contrasted with osteogenic OCN signatures detected in endothelial progenitor cells (EPCs) and vascular lesions. Here, OCN expression aligns with osteogenic drift, immune–calcific bias, and coronary artery calcification (CAC), a phenotype fundamentally different from the circulating endocrine hormone. Figure 7 illustrates this mediator–marker distinction by juxtaposing endocrine ucOCN with osteogenic OCN within EPCs and lesional tissue [159,160,161,162,163].

**Figure 8:**
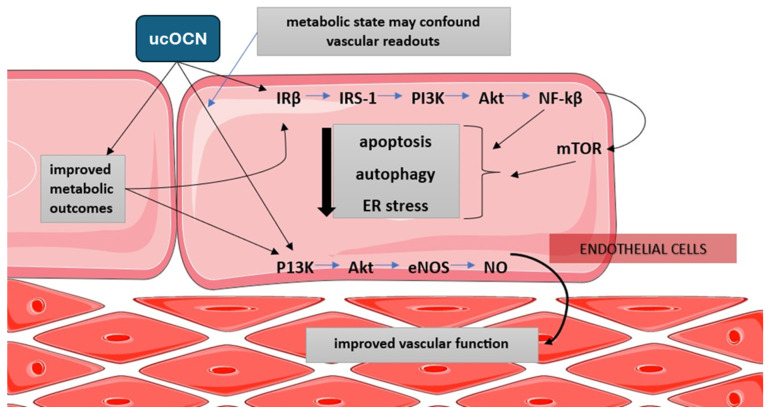
ucOCN signaling pathways in endothelial and vascular smooth muscle cells. The scheme illustrates compartment-specific effects of ucOCN: activation of the IRβ → IRS-1 → PI3K → Akt → eNOS axis in endothelial cells, leading to increased nitric oxide (NO) production and improved endothelial function; and attenuation of NF-κB and mTOR signaling in VSMCs, reducing ER stress, apoptosis, and autophagy. The figure also highlights the influence of systemic metabolic improvements, which may enhance endothelial function independently of ucOCN, underscoring the need to distinguish direct vascular actions from metabolic confounding. Parts of the figure were created using images provided by Servier Medical Art (https://smart.servier.com), licensed under CC BY 4. (https://creativecommons.org/licenses/by/4.0/) (accessed on 7 January 2026).

A concise molecular summary of ucOCN’s endocrine actions in vascular cells is provided in Table 23, which focuses on receptor engagement and core intracellular events without reproducing the full pathway illustrated in Figure 8.

### 6.2. Human Evidence: EPC Biology, Lesional Signatures, and Calcification

Human studies reinforce this compartmental divergence. Circulating ucOCN behaves as a metabolic–endocrine marker with neutral vascular safety, whereas OCN^+^ EPCs and lesional OCN correspond to osteogenic reprogramming and CAC-linked progression [161,162]. Serum OCN levels often correlate inconsistently with angiographic burden, reflecting the biological mismatch between circulating endocrine signals and osteogenic cues in vascular tissue.

Genetic evidence extends this interpretation: Mendelian randomization analyses indicate that variants associated with higher circulating OCN levels are also associated with CAC-mediated coronary artery disease and myocardial infarction risk [163]. These findings strengthen the concept that ucOCN is not a vascular risk molecule, whereas cell-associated OCN behaves as a calcific marker in high-risk states.

### 6.3. Practical Interpretation: Assays, Compartments, Endpoints

Accurate vascular interpretation of OCN demands explicit compartmental awareness. Endocrine actions should be evaluated using bioactive, full-length ucOCN, whereas osteogenic processes should be quantified by OCN^+^ EPCs or lesional staining. Vascular endpoints—flow-mediated dilation (FMD), pulse-wave velocity (PWV), and CAC progression—serve as anchors for interpreting these signals. Vitamin K status and metabolic milieu must also be considered, as both influence the ucOCN/cOCN balance and vascular readouts [14,15,16,17,18,19,147,148,149,150,151,152,153,154,155,156,157,158].

Metabolic interventions such as exercise or weight loss typically increase ucOCN and improve NO-related indices without elevating OCN^+^ EPCs, whereas individuals prone to calcification may follow the opposite trajectory. Adjunctive markers, including dp-ucMGP, AOPPs, and tissue inhibitor of metalloproteinases-1 (TIMP-1), help contextualize vascular remodeling, and systems-biology models provide mechanistic insight into immune-calcific transitions [147,148,149,150,151,152,153,154,155,156,157,158,164].

### 6.4. Oxidative–Immune–ECM Context, Epidemiology, and Integrative Interpretation

Atherosclerosis arises from interacting processes that include oxidative stress, reduced nitric oxide bioavailability, lipid and extracellular matrix disorganization, and immune polarization toward pro-inflammatory states [164,165,166]. Within this complex environment, circulating ucOCN remains vascularly neutral *ex vivo*. It aligns with NO-supportive biology, whereas OCN-positive endothelial progenitor cells and lesional OCN reflect osteogenic, calcification-aligned remodeling within the arterial wall. Genetic and computational studies reinforce this compartment-stratified perspective, showing that endocrine ucOCN and osteogenic OCN signatures map onto distinct biological domains [161,162,163,164,165,166].

From an epidemiological standpoint, cardiovascular disease remains the leading global cause of mortality [167,168,169,170,171,172,173], but in this review, it serves primarily as a translational test case for interpreting osteocalcin biology. Endocrine ucOCN, circulating in blood, aligns with metabolic status and NO-related endothelial tone, whereas OCN^+^ EPCs and lesional OCN correspond to osteogenic drift and coronary-artery calcification. Recognizing this divergence is essential for integrating vascular observations with the broader endocrine, hepatic, metabolic, and oncological axes of OCN physiology.

Human lesion studies provide consistent support for this model. OCN^+^ EPCs and lesional OCN staining correlate more strongly with calcification severity and CAD burden than any circulating OCN form [161,174,175,176]. In contrast, ucOCN behaves as a systemic endocrine marker rather than a vascular risk molecule. Distinguishing endocrine ucOCN in circulation from osteogenic OCN in EPCs or lesions, therefore, provides a pragmatic interpretive scaffold for vascular research and clinical study design.

Future cardiometabolic studies should quantify bioactive ucOCN alongside OCN^+^ EPCs fractions and vascular endpoints—flow-mediated dilation, pulse wave velocity, and coronary calcium progression—while adjusting for metabolic state and vitamin K status. Only such compartment-aware approaches can disentangle endocrine signaling from osteogenic remodeling and clarify how these trajectories evolve under metabolic or vascular pressure.

### 6.5. Diet, Vitamin K2, and Vascular Implications

Diet modulates vitamin K_2_ intake and, in turn, OCN carboxylation. Plant-based dietary patterns—particularly vegan diets—are characterized by very low menaquinone intake, which may elevate %ucOCN due to limited exposure to MK-7 and MK-4, as confirmed in independent nutritional assessments and clinical reviews [177,178,179,180,181,182]. In contrast, fermented foods (notably natto) and selected animal-derived products provide meaningful sources of long-chain and short-chain menaquinones, consistent with current biochemical overviews of diet-derived K_2_ forms. In dialysis populations, MK-7 supplementation lowers %ucOCN and dp-ucMGP but does not guarantee regression of vascular calcification [147,148]. Dietary patterns are compared in Table 24.

### 6.6. Synthesis and Methodological Considerations

Current data support a dual interpretation of osteocalcin in the vascular system. At physiological concentrations, circulating ucOCN behaves as an endocrine mediator that is vascularly safe, supports NO bioavailability, and aligns with systemic metabolic benefits [159,160]. In contrast, OCN^+^ EPCs and lesional OCN in high-risk vascular settings reflect an osteogenic phenotype, consistent with calcific drift and with genetic evidence linking elevated OCN to CAC-mediated atherogenesis [161,162,163]. Treating ucOCN as a circulating hormone and OC expression on vascular or progenitor cells as an osteogenic marker is therefore essential for conceptual clarity.

The next phase of research should test these two trajectories within the same individuals by jointly quantifying circulating ucOCN, OCN^+^ EPCs, and vascular outcomes, including CAC, FMD, and PWV. Such a design would clarify whether endocrine and osteogenic signatures evolve in parallel or diverge under metabolic pressure [160,161]. Causal replication requires refining Mendelian instruments to ucOCN-specific variants rather than total OCN proxies, and validating CAC mediation across multiple calcification GWAS datasets [163]. Mechanistically, metabolic interventions, such as exercise, should elevate ucOCN and improve NO indices without increasing OCN^+^ EPCs^+^, whereas subjects with progressive calcification may show discordant patterns [159]. This compartment-aware interpretation—hormone in circulation, marker in plaques—provides a coherent basis for translational trial design.

Interpreting these relationships depends heavily on methodological precision. Most existing human datasets rely on total OCN or N-MID assays, which aggregate fragments and carboxylation states and may obscure ucOCN-specific biology. Only full-length ucOCN, as measured by validated immunoassays or LC–MS, captures endocrine signaling relevant to vascular physiology. Just as critical is the anatomical compartment: circulating ucOCN aligns with PI3K/Akt/eNOS-linked endothelial signaling, whereas OC expressed on EPCs or vascular cells reflects osteogenic, calcification-aligned remodeling [161,162,176]. Without separating these domains, contradictory associations with CAD and calcification indices are unsurprising.

Finally, improvements in NO biology may reflect systemic insulin sensitization rather than a direct vascular effect of ucOCN, underscoring the need to co-measure metabolic and vascular endpoints to prevent misattribution [159,174]. Mechanistic foundations summarized in Table 23 and Table 25 anchor this interpretive framework.

These relationships are illustrated schematically in Figure 8.

### 6.7. Practical Framework for Vascular OCN Trials

A rigorous evaluation of vascular osteocalcin biology requires a framework that distinguishes the endocrine actions of circulating ucOCN from the osteogenic signatures detected within vascular or progenitor compartments. These two signaling modes often coexist, yet they reflect fundamentally different pathophysiological domains. Clinical trials must therefore adopt a compartment-aware design to accurately interpret OCN-related effects.

Individuals with insulin resistance or T2DM, as well as those with early vascular alterations such as increased pulse-wave velocity or detectable coronary calcium, constitute the most informative study populations. These groups provide the necessary metabolic–vascular contrast to separate ucOCN-related endocrine phenomena from osteogenic reprogramming within vascular tissues.

To ensure interpretive clarity, endocrine and vascular endpoints must be assessed concurrently. Bioactive ucOCN should be measured using LC–MS or validated full-length immunoassays, as total OCN introduces analytical noise and may obscure ucOCN-specific associations [15,16,17,18]. In parallel, quantification of OCN^+^ EPCs serves as a readout of osteogenic drift within the vasculature. At the same time, vascular function should be characterized using established indices such as flow-mediated dilation, pulse wave velocity, coronary calcium progression, and markers of NO bioavailability.

Interventional components—exercise, vitamin K status, or metabolic therapies—must be chosen and interpreted with attention to the compartment they influence. Exercise engages the bone–muscle–vascular axis and typically elevates ucOCN, whereas vitamin K supplementation modifies ucOCN–cOCN balance without directly informing endothelial biology. Improvements in NO signaling may mirror enhanced insulin sensitivity rather than reflect direct endocrine action; thus, metabolic endpoints must be co-measured to avoid misinterpretation [142,157]. Conversely, vascular calcification or osteogenic remodeling should be inferred primarily from OCN^+^ EPCs fractions or CAC progression, not from serum OCN concentrations alone [144,145,159].

Transparent reporting is essential. Each study must specify the analyte measured (tOCN, N-MID, full-length ucOCN), assay platform, sample-handling conditions, vitamin K exposure, renal status, metabolic profile, and vascular readouts. Without such detail, comparisons across studies become unreliable, and compartment-specific signals risk being conflated.

Ultimately, methodological precision—assay, analyte, and compartment—determines whether an OCN study advances mechanistic understanding or reinforces existing ambiguity. A coherent framework that integrates these elements is required to accurately delineate the vascular relevance of OCN.

## 7. OCN at the Bone–Tumor Interface

### 7.1. Epidemiological Context and Compartment-Aware Framework

OCN participates in tumor biology in two fundamentally different ways, depending on the compartmental context. As cancer incidence continues to rise globally—with approximately 20 million new cases and 9.7 million deaths in 2022 [183], 2.7 million new diagnoses annually across the EU-27 [184], and national Polish estimates of ~208,900 new cases and 119,992 deaths in 2022 [185,186]—these distinctions are increasingly relevant for understanding tumor–bone crosstalk and metastatic progression.

Within this epidemiological backdrop, OCN operates through two complementary yet distinct roles. First, as an endocrine mediator, circulating ucOCN can interact with receptors expressed on tumor cells, including the GPRC6A axis in prostate cancer and the transforming growth factor-β (TGF-β)/mothers against decapentaplegic homolog 3 (SMAD3) pathway in triple-negative breast cancer (TNBC). These interactions have been mechanistically linked to proliferative, metabolic, EMT-related, and osteolytic programs [187,188,189]. Second, as osteogenic markers, circulating OCN^+^ EPCs and lesional OCN reflect stromal remodeling, pre-metastatic niche (PMN) conditioning, and bone-tropic engagement—processes particularly evident in breast cancer metastasis [190,191,192,193]. Here, OCN acts less as a hormone and more as a readout of microenvironmental osteogenic drift.

Recognizing this duality—ucOCN as an endocrine signal versus cell-associated OCN as an osteogenic marker—is critical for interpreting tumor biology at the bone interface.

To orient this framework, Table 26 summarizes the compartment-aware perspective, contrasting circulating ucOCN–receptor interactions (e.g., GPRC6A; TGF-β/SMAD3) with osteogenic OCN signatures associated with PMN conditioning and bone metastasis [187,188,189,190,191,192,193].

### 7.2. OCN as a Tumour Mediator: Receptors and Pathways

OCN acts as a direct tumor-modulating signal in selected malignancies through receptor-specific pathways. The best-established endocrine mediator axis involves GPRC6A, which is expressed on prostate cancer cells and integrates OCN/ucOCN signaling into proliferative and metabolic programs. Experimental studies demonstrate that OCN/ucOCN activates GPRC6A-dependent signaling through extracellular signal-regulated kinase (ERK), Akt, and mTOR, promoting tumor growth in vitro and in xenograft models [187,188]. CRISPR/Cas9 gene-editing system (clustered regularly interspaced short palindromic repeats/CRISPR-associated protein 9)-mediated deletion of GPRC6A suppresses these pathways and markedly reduces tumorigenicity, confirming that the OCN–GPRC6A axis is functionally relevant in prostate cancer [187,188].

In TNBC, circulating ucOCN can also function as a tumor mediator via a distinct signaling pathway. Here, ucOCN enhances TGF-β/SMAD3 activity, driving epithelial–mesenchymal transition (EMT), matrix remodeling, angiogenic signaling, and osteolytic programming, all characteristic of bone-tropic metastatic behavior [189]. These findings indicate that ucOCN, depending on the tumor’s receptor landscape, may potentiate invasiveness and metastatic potential.

Together, these endocrine interactions support a model in which ucOCN functions as a receptor-engaged tumor mediator, leveraging GPRC6A or TGF-β/SMAD3 pathways to promote distinct malignant phenotypes across tumor types. Mechanistic details and phenotypic outputs for prostate cancer and TNBC are summarized in Table 27.

### 7.3. Biomarker Axis: Early Tracking of Bone Metastatic Risk

Clinical evidence shows that osteocalcin contributes to the early detection of bone-tropic disease through osteogenic cellular signatures, not solely through endocrine signaling. In breast cancer cohorts, the proportion of OCN^+^ circulating EPCs increases in patients with bone involvement and can discriminate progressive from stable skeletal disease before radiologic progression is detectable [190]. This positions OCN^+^ EPCs as a promising biomarker for early bone-metastasis (BM) risk stratification in cancers with strong skeletal tropism.

Lesional analyses further demonstrate that OCN expression within the bone microenvironment reflects osteogenic drift, stromal remodeling, and pre-metastatic niche (PMN) conditioning. Spatial profiling studies reveal that OCN-rich microenvironments coincide with matrix reorganization, osteoblast–osteoclast crosstalk, and immune modulation that precede overt metastatic colonization [191,192,193].

Together, these findings indicate that osteogenic OCN signatures (OCN^+^ circulating EPCs, lesional OCN staining) act as markers of PMN activation and early metastatic dynamics—distinct from circulating ucOCN, which functions as an endocrine tumor mediator (see Section 7.2). Table 28 summarizes these biomarker-level insights across bone-tropic malignancies.

### 7.4. Compartment-Aware Study Design: Endocrine vs. Osteogenic Readouts

A compartment-aware design framework is essential for correctly interpreting osteocalcin-related signals in malignancy. Because circulating ucOCN (an endocrine mediator) and cOCN/lesional OCN (an osteogenic marker) represent biologically distinct compartments, studies exploring tumor progression, metastatic patterning, or treatment response must evaluate both axes in parallel. This dual-compartment approach aligns with the mechanistic and biomarker findings outlined previously (Section 7.1, Section 7.2 and Section 7.3).

From an endocrine standpoint, bioactive ucOCN should be measured using assays that differentiate ucOCN from total OCN, with explicit documentation of vitamin K status and assay characteristics. This is crucial for assessing endocrine effects mediated through GPRC6A or TGFβ/SMAD3, as described for prostate cancer and TNBC [187,188,189].

From an osteogenic marker perspective, cOCN-positive circulating cells and lesional OCN staining indicate osteogenic reprogramming, PMN activation, and bone-tropic behavior, supported by translational breast cancer data [190,191,192,193].

Because these compartments capture different biological processes, clinical research must harmonize sampling, measurement, and timing to avoid misinterpretation. A practical structure integrating both endocrine ucOCN and osteogenic cOCN/lesional OCN is provided in Table 29, which outlines baseline phenotyping, on-therapy monitoring, mechanistic layers, and imaging endpoints.

### 7.5. Interpreting ucOCN: Carboxylation and Calcification Signals

Because ucOCN reflects the degree of γ-carboxylation, its interpretation in oncology must consider vitamin K status, which shapes both endocrine signaling and vascular/calcification biology. A systematic review and meta-analysis show that vitamin K supplementation can slow vascular calcification and reduce dp-ucMGP, whereas higher dp-ucMGP levels associate with adverse cardiometabolic profiles [196,197]. Accordingly, ucOCN assessment in tumor cohorts should document dietary vitamin K intake, supplementation, or antagonism to avoid misclassification of endocrine activity.

In contrast, tissue-level OCN immunohistochemistry offers limited diagnostic utility in bone tumors. Contemporary pathology demonstrates that alkaline phosphatase (ALP) outperforms OCN for distinguishing osteosarcoma from other primary bone malignancies, reflecting superior tissue specificity and robustness [198]. Thus, although ucOCN may act as an endocrine mediator in prostate cancer or TNBC (Section 7.1 and Section 7.2), and OCN-positive circulating EPCs may track bone-tropic disease (Section 7.3), lesional OCN staining should not be used as a standalone diagnostic marker.

Taken together, ucOCN is best viewed as a functional readout of vitamin K-dependent γ-carboxylation, while tissue OCN positivity reflects osteogenic microenvironmental remodeling rather than endocrine signaling. These distinctions are essential for correctly situating OCN readouts in oncologic research, particularly in bone-prone malignancies or studies including vascular/calcification endpoints [196,197,198].

### 7.6. Synthesis and Translational Outlook

OCN occupies a unique position at the crossroads of endocrine signaling and osteogenic microenvironmental remodeling in cancer. The evidence summarized across Section 7.1, Section 7.2, Section 7.3, Section 7.4 and Section 7.5 highlights two biologically and translationally distinct axes through which OCN operates.

On the endocrine side, circulating ucOCN can engage tumor-expressed receptors such as GPRC6A in prostate cancer and TGF-β/SMAD3 in TNBC, activating proliferative, metabolic, and EMT-linked programs, as well as osteolytic signaling pathways [187,188,189]. These interactions suggest that, in tumors responsive to bone-derived cues, ucOCN may amplify malignant behavior via receptor-level signaling.

In parallel, osteogenic OCN signatures—reflected by OCN-positive circulating EPCs and lesional OCN within the tumor–bone microenvironment—align with stromal remodeling and PMN activation. Rising OCN^+^ EPC fractions correspond to emerging skeletal involvement in breast cancer, while lesional OCN marks niches undergoing osteogenic drift and early bone-tropic conditioning [190,191,192,193]. In this setting, OCN serves not as an endocrine driver but as an indicator of microenvironmental engagement and metastatic potential. Notably, tissue-level OCN staining should not be misinterpreted diagnostically in primary bone tumors; alkaline phosphatase (ALP) remains the more reliable discriminant in osteosarcoma evaluation [198].

Taken together, these data demonstrate that meaningful translational use of OCN requires a compartment-aware strategy. Circulating ucOCN and osteogenic OCN^+^ EPCs/lesional OCN capture fundamentally different biological signals and must therefore be interpreted accordingly. Clinical studies should measure circulating ucOCN to interrogate endocrine pathways, assess OCN-positive circulating EPCs and lesional expression to monitor osteogenic niche activity, stratify findings by receptor-level context (e.g., GPRC6A, TGF-β/SMAD3), and document vitamin K status to interpret ucOCN dynamics correctly.

Within such a structured framework, OCN serves as both a mechanistic mediator and a readout of microenvironmental remodeling, providing a coherent basis for future diagnostic and therapeutic advances in bone-tropic malignancies [187,188,189,190,191,192,193,198]. Adjacent microenvironmental signaling axes relevant to this translational landscape are summarized in Table 30.

## 8. Natural Modulators of Osteocalcin: From Diet to Therapeutics

OCN is shaped by nutritional, microbial, and lifestyle inputs that jointly influence its transcription, post-translational γ-carboxylation, and downstream endocrine or matrix-linked signaling. These natural modulators extend beyond classical bone metabolism, integrating skeletal, metabolic, and vascular physiology and providing a translational framework illustrated in Figure 3.

### 8.1. Dietary Polyphenols as Upstream Modulators

Dietary polyphenols act as broad upstream regulators of the cellular milieu in which osteocalcin operates. Through antioxidant and anti-inflammatory actions, they reduce redox stress, modulate immune tone, and support endothelial signaling pathways that intersect with OCN-dependent bone–vascular physiology [201]. Complementary small-molecule studies demonstrate that polyphenol-derived scaffolds exert anti-inflammatory and anti-diabetic effects, aligning with the systems-level perspective developed in this review [202]. Together, these observations position polyphenols as indirect yet meaningful modulators of OCN biology by enhancing the metabolic and endothelial conditions in which both endocrine and matrix-embedded OCN exert their functions.

### 8.2. Vitamins D_3_ and K_2_—Synergistic Modulation

Vitamin D_3_ and vitamin K_2_ act at complementary mechanistic tiers of the osteocalcin pathway. Vitamin D_3_ increases *BGLAP* transcription and promotes osteoblast maturation, expanding the pool of total OCN [66,68]. Vitamin K_2_, functioning as the essential cofactor for GGCX, enables γ-carboxylation and conversion of OCN into its mineral-binding form (cOCN) [66,67,68,69]. This D_3_-driven synthesis and K_2_-dependent activation form the biochemical foundation of functional osteocalcin.

Clinical data consistently show that K_2_ supplementation shifts OCN toward its carboxylated fraction, lowering circulating ucOCN and modestly improving skeletal outcomes. Long-term MK-7 intake (180–375 µg/day) reduces ucOCN and enhances indices of bone strength, while MK-4 lowers ucOCN at pharmacologic doses without short-term gains in BMD [73,74,75,76,77]. Beyond bone, D_3_ and K_2_ intersect with vascular physiology: adequate K_2_ supports the activation of both OCN and matrix Gla protein (MGP), reinforcing endothelial nitric oxide-based homeostasis [68,69]. In postmenopausal cohorts, MK-7 supplementation has also been associated with attenuated arterial stiffness and favorable blood pressure trends [33,203].

Overall, these nutrients converge to enhance both skeletal and vascular dimensions of OCN biology. Table 31 summarizes this integrated landscape of upstream modulators.

### 8.3. Dietary and Microbiome-Directed Modulation of the K_2_–OCN Axis

Mediterranean and plant-forward dietary patterns, characterized by abundant polyphenols, vegetables, legumes, olive oil, and fermented foods, create a biochemical environment that supports osteoblast function and endothelial health. By improving redox balance, reducing inflammation, and enhancing NO-dependent signaling, these diets reinforce molecular pathways that influence OCN synthesis and activity [69,201,204].

A central mechanistic link to OCN activation is the microbiome-derived synthesis of menaquinones (MK-n). Bacterial genera such as *Bacillus* and *Bifidobacterium* produce MK-7 and related menaquinones, which contribute to endogenous vitamin K_2_ supply and support γ-carboxylation of OCN [65,66]. Dietary fiber, fermented foods, and prebiotic substrates indirectly enhance the OCN axis by shaping microbiome composition and menaquinone output.

Probiotic and microbiome-directed strategies extend this nutritional synergy. *LactoBacillus/LacticaseiBacillus*, *Bifidobacterium*, and *Bacillus* species influence short-chain fatty acid (SCFA) production, epithelial barrier integrity, and cytokine tone, thereby shaping an immunometabolic environment that favors vitamin K-dependent protein activation [63,64]. *Bacillus* spp. are of particular translational interest: selected strains synthesize MK-7 within the gastrointestinal tract, reinforcing γ-carboxylation capacity for OCN activation [65]. Strain-level characterization, including *Bacillus* subtilis isolated from fermented foods, aligns this probiotic potential with dietary MK-7 sources and supports combined dietary–microbial interventions [205].

Although clinical data are heterogeneous, probiotic supplementation yields small but significant improvements in lumbar spine BMD over 6–12 months, particularly in postmenopausal women, whereas responses in hip BMD vary [188]. Collectively, these findings show that dietary patterns, microbiome-derived menaquinones, and probiotic approaches form an integrated nutritional–microbial strategy that supports endogenous MK-n production, enhances OCN carboxylation potential, and coordinates bone–vascular endocrine physiology [63,64,65,69,201,204,205]. Mechanistic examples are summarized in Table 32.

### 8.4. Context-Dependent Roles in Oncology

OCN signaling in oncology is context-dependent and shaped by tumor receptor profiles and microenvironmental cues. In TNBC, ucOCN activates the TGF-β/SMAD3 axis, enhancing EMT-like, osteolytic, and bone-tropic behaviors [189]. In prostate cancer, ucOCN interacts with GPRC6A, activating MAPK/mTORC1 signaling and revealing a receptor-specific mechanism distinct from TNBC pathways [187,188].

Clinically, OCN^+^ EPS circulating or progenitor cells have been explored as biomarkers of bone-metastatic progression, particularly in breast cancer, where they mirror early skeletal involvement and PMN conditioning [190,191,192,193]. These osteogenic signatures reflect microenvironmental remodeling rather than endocrine signaling, linking the OCN axis to stromal and extracellular vesicle networks that modulate metastatic readiness [191,192,193].

Overall, OCN integrates phenotype-specific endocrine and osteogenic signals rather than exerting uniform pro- or anti-tumor effects across malignancies [187,188,189,190,191,192,193].

### 8.5. From “Lipidogram” to Endothelium: HDL Functionality and NO Biology

Recent mechanistic frameworks emphasize HDL functionality—particularly cholesterol efflux capacity—and nitric oxide-centered endothelial signaling, as key determinants of vascular health, surpassing the relevance of static lipid concentrations [206]. Within this framework, dietary and micronutrient inputs, such as polyphenol-rich Mediterranean diets and vitamin K-dependent protein activation, intersect with eNOS pathways that support vascular integrity [207,208].

Because OCN interacts with both metabolic and vascular systems, adequate vitamin K status facilitates γ-carboxylation of OCN and MGP, stabilizing vascular redox balance and modulating NO bioavailability [69]. This integration positions the K_2_–OCN axis alongside HDL functional metrics as part of a broader re-definition of cardiometabolic risk assessment [206,207,208].

### 8.6. Translational Implications and Future Design

Translational strategies targeting the OCN axis require harmonized measurement and phenotype-specific interpretation. Clinical studies should differentiate tOCN from N-MID and ucOCN/cOCN, integrate dp-ucMGP as a surrogate of vitamin K status, and align skeletal, metabolic, and vascular endpoints within unified protocols [5,8,69]. Combination interventions—such as MK-7 supplementation (enhancing γ-carboxylation), polyphenol-rich diets (supporting endothelial and redox tone), and exercise (activating OCN–muscle crosstalk)—offer biologically coherent approaches for future trials [4,73,201].

Given the requirement for GLP-1 receptor signaling in several OCN-linked metabolic effects in preclinical models, human studies incorporating incretin endpoints or receptor blockade could further clarify mechanistic specificity [143,144]. Ultimately, robust trial design must be assay-aware, vitamin K-aware, and phenotype-stratified to capture the interconnected skeletal, metabolic, and vascular dimensions of osteocalcin biology [5,14,17,69,138,139,140,141,142,143,144].

### 8.7. Updated Preclinical Consensus

A recent methodological evaluation integrates multiscale evidence to clarify osteocalcin’s biological roles, resolving inconsistencies stemming from earlier knockout studies. Contemporary validation demonstrates that OCN influences bone material properties, including mineral crystal size, alignment, and matrix organization—beyond its contribution to BMD. Updated metabolic analyses further show that OCN participates in glucose regulation through coordinated skeletal remodeling, insulin sensitivity, and energy homeostasis. These insights suggest that prior discrepancies in *Ocn*^−/−^ phenotypes reflect strain variability, analytical limitations, and incomplete recognition of compensatory pathways, offering a consolidated mechanistic framework for modern OCN biology [33].

## 9. Integrative Conceptual Framework and Translational Implications

OCN emerges as a systems-level coordinator linking skeletal remodeling with whole-body energy allocation. Within this integrative framework, insulin signaling in osteoblasts establishes a remodeling milieu that releases bioactive ucOCN. Once in circulation, ucOCN engages its key receptors—GPRC6A and GPR158—to tune peripheral and central physiology, aligning metabolic, neuromuscular, reproductive, and cognitive demands with fuel availability (see Section 5.2 and Section 5.3) [11,12,15].

Interpretation of these pathways depends critically on analytical precision. N-MID reflects bone turnover dynamics, whereas full-length ucOCN captures endocrine signaling capacity; conflating these analytes can obscure physiological meaning. This receptor-anchored distinction is essential, as detailed in Section 5.2 and Section 5.3.

Human data are most consistent where physiology and measurement converge. In middle-aged and older adults, ucOCN correlates with insulin sensitivity and β-cell function, and clamp studies provide modest yet reproducible mechanistic support. By contrast, large prospective cohorts often yield null associations when assays, phenotyping strategies, or population characteristics differ. These discrepancies highlight the need for standardized ucOCN phenotyping and stratification by vitamin K status, adiposity, and receptor biology rather than universalized interpretations [17,93,94,95].

Beyond glucose regulation, preclinical work identifies a robust enterohepatic arm. Via hepatic GPRC6A, ucOCN activates Nrf2, suppresses JNK, and reduces lipogenesis through AMPK-mediated downregulation of SCD1. Synthetic OCN analogues complement these effects by limiting CD36-dependent lipid uptake and improving histological features of NAFLD. A portion of this metabolic benefit requires GLP-1 signaling, positioning ucOCN within a gut–liver axis relevant to glucose tolerance and hepatic resilience [138,139,140,141,143,144,146].

A second extension involves the bone–testis axis. ucOCN binds GPRC6A on Leydig cells, enhancing steroidogenesis in parallel to the hypothalamic–pituitary–gonadal (HPG) system. Human genetic evidence involving GPRC6A variants and cell-based models supports this mechanism. Conversely, total OCN generally reflects HPG-driven bone turnover rather than endocrine ucOCN activity, underscoring the need to match the analyte to the biological question [84,113].

Neurocognitive and stress-response pathways provide further illustration of OCN’s systems-level role. Acting through GPR158 in the brain, ucOCN enhances memory, reduces anxiety-like behavior, and supports neurometabolic coupling. During acute stress, circulating bioactive OCN rises within minutes and modulates autonomic balance, consistent with a rapid bone-derived contribution to the acute stress response [3,12,121,127,136].

Taken together, this receptor-anchored, compartment-aware framework supports a unified approach to OCN measurement. Use N-MID to assess bone turnover; use full-length ucOCN—paired with vitamin K phenotyping—when interrogating endocrine or metabolic pathways. Observational signals in the liver–gut, reproductive, and brain/stress axes must be interpreted within this analytical scaffold rather than as isolated findings [5,14,17,138,139,140,141,142,143,144]. This integrated perspective consolidates osteocalcin’s role as a biochemical nexus linking metabolic, skeletal, and neuroendocrine systems.

## 10. Limitations

This narrative review integrates findings generated across heterogeneous analytical platforms, study designs, and biological systems. Interpretation of human data is particularly constrained by assay choice, as tOCN, N-MID, and full-length ucOCN capture fundamentally different physiological signals and are variably influenced by circadian timing, vitamin K status, and renal function. These analytical distinctions complicate cross-study comparisons and limit the generalizability of aggregate conclusions.

Preclinical evidence further carries important caveats. Knockout models exhibit strain-specific metabolic and skeletal phenotypes, and differences in assay methods, dietary K status, or compensatory pathways complicate direct translation to humans. Moreover, endocrine and osteogenic OCN signals are frequently conflated across studies, obscuring compartment-specific biology.

Finally, as this is a narrative rather than a systematic review, search methods were not exhaustive, and conclusions should be interpreted qualitatively and within the context of available evidence. These limitations underscore the importance of the methodological and analytical considerations elaborated in Section 11.

## 11. Future Directions

Translating OCN biology into clinical practice requires a harmonized approach that integrates analytical precision, physiological variability, and organ-specific context. Much of the apparent controversy in the OCN literature arises from inconsistent assay use, strain-dependent divergence in knockout models, and phenotype-specific variation in human cohorts [5,10,14]. A coherent future roadmap must therefore rest on methodological rigor, combined with receptor-anchored interpretation and state-dependent stratification.

Analytical foundations remain central. Human conclusions about OCN are strongly conditioned by assay choice: N-MID provides a stable index of bone turnover, whereas full-length ucOCN captures endocrine and metabolic signaling. These analytes are not interchangeable, and their interpretation must account for circadian timing, pre-analytical handling, vitamin K status, and renal function, all of which modulate the ucOCN/cOCN balance and can confound epidemiological findings [5,10,14,17,18]. Without explicit reporting of analyte type (tOCN, N-MID, ucOCN), assay platform, sampling times, and vitamin K exposure, cross-study synthesis remains inherently heterogeneous.

Where physiology and analytics converge, human studies consistently show modest but reproducible links between ucOCN and insulin sensitivity or secretion. However, observational meta-analyses exhibit small effect sizes and considerable heterogeneity, and large prospective datasets often yield null associations when assays, phenotyping strategies, or endpoints diverge [17,93,94,95]. Mendelian-randomization analyses further illustrate the complexity of bone–glucose interactions: genetically elevated bone mineral density is associated with increased T2DM risk, indicating that OCN is not the sole driver of bone–metabolism crosstalk [97,98]. These findings emphasize the need for phenotype-aware and assay-aware interpretation rather than universal conclusions.

Future clinical studies will require stratification by vitamin K status, adiposity, and, where feasible, receptor biology such as GPRC6A or GPR158 expression. ucOCN should be predefined as the mechanistic readout in metabolic studies, while tOCN or N-MID may serve complementary roles in skeletal contexts. Early intervention trials already demonstrate strong state-dependence: combined vitamin D_3_ and K_2_ improves glycemic markers in T2DM, whereas long-term MK-7 lowers ucOCN and increases adiponectin without improving HOMA-IR in healthy women [75,77]. The requirement of GLP-1 signaling for several preclinical ucOCN effects suggests that human trials incorporating incretin endpoints or GLP-1R blockade may help to clarify mechanistic specificity [143,144].

Beyond glucose regulation, OCN participates in multiple organ axes. In the liver–gut axis, ucOCN engages hepatocellular GPRC6A, activates Nrf2, suppresses JNK, downregulates SCD1 through AMPK pathways, and improves NAFLD phenotypes, while synthetic OCN analogues reinforce these effects by limiting CD36-mediated lipid uptake [138,139,140,141,142]. In the reproductive axis, ucOCN acts on Leydig cells via GPRC6A, whereas total OCN primarily reflects HPG-driven bone turnover rather than endocrine bioactivity [84,113]. In the brain and stress axes, ucOCN–GPR158 signaling enhances memory, reduces anxiety-like behavior, and rapidly modulates the autonomic nervous system during acute stress [3,12,121,126,136]. These organ-specific extensions underscore the value of a receptor-anchored scaffold for interpreting disparate physiological readouts.

Safety considerations are equally important. Renal function profoundly modifies OCN clearance: intact and fragmented OCN accumulate as GFR declines, requiring nephrology-specific interpretative frameworks in CKD–MBD, including paired measurements of PTH, BALP, intact PINP, and TRACP-5b [147,148,149,150,151]. Vitamin K insufficiency is highly prevalent in dialysis and elevates %ucOCN and dp-ucMGP; although MK-7 improves carboxylation markers, vascular outcomes remain inconsistent [152,153,154,155,156]. Vitamin K antagonists distort ucOCN fractions, and intact OCN remains pre-analytically unstable unless measured as N-MID [8,25].

A future-ready framework will therefore require explicit documentation of OCN analyte, sampling conditions, vitamin K exposure, renal function, metabolic state, and organ-specific endpoints such as GLP-1, hepatic imaging, reproductive hormones, cognitive tasks, or stress paradigms—while accounting for concurrent therapies that influence bone turnover or K-dependent proteins [5,10,14,17,143,144,147,148,149,150,151]. Such analytically precise and phenotype-stratified methodology provides a coherent foundation for advancing osteocalcin biology across metabolic, vascular, hepatic, reproductive, and neurocognitive axes.

## 12. Summary and Conclusions

A cohesive body of recent evidence indicates that many longstanding controversies in osteocalcin research can be traced to methodological inconsistencies, assay heterogeneity and strain-dependent phenotypes in early knockout studies. Contemporary reassessment integrates these findings into a unified framework in which OCN contributes to both bone material quality and whole-body metabolic control [38]. Within this structure, N-MID remains the analyte of choice for bone-turnover assessment. In contrast, full-length ucOCN, interpreted alongside vitamin K status, circadian timing, and renal function, is required to capture endocrine and metabolic actions [5,14,17,138,139,140,141,142,143,144].

Across physiological systems, OCN has transitioned from a skeletal turnover marker to a systems-level hormone linking bone remodeling with metabolic, hepatic, reproductive, neurocognitive, and stress response pathways. Bioactive ucOCN acts through GPRC6A and GPR158 to modulate insulin secretion and sensitivity, muscle glucose uptake, Leydig-cell steroidogenesis, hippocampal plasticity, and rapid autonomic adjustments during acute stress. Preclinical work further supports a hepatoprotective enterohepatic arm—characterized by Nrf2 activation, JNK suppression, and AMPK-mediated downregulation of SCD1—and highlights GLP-1 and Piezo1 signaling as additional mechanistic nodes requiring human validation.

Vascular and oncological data reinforce a compartment-aware interpretation: circulating ucOCN functions as an endocrine hormone, whereas OCN embedded within EPCs or tumor-associated lesions reflects osteogenic or calcific remodeling rather than systemic signaling. These distinctions are essential for translational clarity.

Looking forward, progress will depend on standardized ucOCN phenotyping, vitamin K-aware trial design, and receptor-anchored mechanistic frameworks incorporating GPRC6A and GPR158 biology. Lifestyle-based strategies—including exercise, polyphenol-rich dietary patterns, and K_2_ supplementation—offer biologically coherent interventions for future studies. Ultimately, OCN emerges as a systems integrator of metabolic, endocrine, and skeletal homeostasis, whose clinical utility will require harmonized analytics and context-specific human validation.

This integrative perspective is captured in the graphical abstract, which synthesizes the compartment-aware OCN framework—from osteoblast-driven generation and receptor-anchored endocrine signaling to tissue-embedded osteogenic signatures—into a unified model of skeletal, metabolic, vascular, and neuroendocrine homeostasis.

## Figures and Tables

**Table 1:** Structural and Functional Differences Between cOCN and ucOCN.

Characteristic	cOCN	ucOCN
Gla residues	3	<3
Calcium binding	High	Low
Localization	Bone matrix	Circulation
Receptors	—	GPRC6A, GPR158, GPR37
Function	Structural role	Endocrine regulation

Abbreviations: cOCN—carboxylated osteocalcin; Gla—γ-carboxyglutamic acid, GPR37—G protein-coupled receptor 37; GPR158—G protein-coupled receptor 158; and GPRC6A—G protein-coupled receptor class C group 6A; ucOCN—undercarboxylated osteocalcin.

**Table 2:** Analytical comparison: BioLegend 8H4/4B6 ELISA vs. Takara Glu-OCN EIA, based on [18,19].

Feature	BioLegend ucOCN ELISA (Capture 8H4/Detection 4B6)	Takara Glu-OC EIA (MK118)
Primary target	Full-length ucOCN	Undercarboxylated OCN epitope (Glu-OC); may detect OCN fragments
Antibody strategy	Sandwich ELISA; capture C-terminal OCN (8H4), detection mid-region ucOCN 12–28 (4B6)	Competitive/indirect EIA against Glu-OC epitope
Analytical range	0.037–1.8 ng/mL (polynomial fit from standards)	0.25–8.0 ng/mL (kit manual)
Limit of detection (LoD)	Approximately 0.015 ng/mL	0.25 ng/mL
Intra-/inter-assay CV	3.6%/7.6%	4.6%/5.7%
Sample volume	<15 µL (effective; diluted input 50 µL per well)	100 µL per determination
Matrix compatibility	Serum; plasma (EDTA, heparin, citrate); good linearity with dilution	Serum/plasma
Specificity/cross-reactivity	Low cross-reactivity with cOCN and partially carboxylated OCN; recognizes pro-ucOCN (limited)	Epitope may bind OCN fragments, potentially overestimating ucOCN.
Functional validation	Vitamin K1 treatment decreases (2×) ucOCN secretion; warfarin in humans increases (4×) ucOCN	Historical fracture-risk studies (EPIDOS) and observational data; no clamp validation reported
Clinical correlations	Stronger, clamp-validated associations with insulin sensitivity, glucose metabolism, and β-cell function compared with tOCN	Associations with insulin sensitivity are often weaker or inconsistent versus direct ucOCN ELISA
Key limitation	Cross-reacts with pro-ucOCN (relevant in cell culture supernatants)	Fragment detection may inflate values and obscure metabolic associations

Abbreviations: β-cell—pancreatic beta cell; cOCN—carboxylated osteocalcin; CV—coefficient of variation; ELISA—enzyme-linked immunosorbent assay; EIA—enzyme immunoassay; EPIDOS—Epidemiology of Osteoporosis Study; Glu-OC—glutamic acid osteocalcin epitope; LoD—limit of detection; OCN—osteocalcin; pro-ucOCN—precursor undercarboxylated osteocalcin; tOCN—total osteocalcin; ucOCN—undercarboxylated osteocalcin.

**Table 3:** Clinical utility of OCN-derived markers (ucOCN/tOCN, N-MID, OC22).

OCN-Derived Markers	Clinical Role	Advantages	Limitations	Availability	References
ucOCN/tOCN ratio	Fracture risk prediction in older adults	Strong independent predictor of fractures; reflects vitamin K status and bone quality.	Limited use in routine practice; requires specialized assays; mainly research-based	Mostly research settings	[6,7]
N-MID OCN fragment	Bone turnover monitoring	High stability; standardized assays; widely used in clinical labs; useful for therapy monitoring	Does not directly predict fracture risk; it is influenced by renal function and circadian variation.	Routine clinical practice	[5,8]
OC22 peptide	Emerging therapeutic target in bone mineralization	Specific bioactive domain; regulates hydroxyapatite formation; potential drug target	Not yet validated in clinical assays; limited to experimental models	Preclinical research only	[21]

Abbreviation: N-MID—N-terminal/mid-region osteocalcin fragment; OC22—osteocalcin 22-residue bioactive peptide; tOCN—total osteocalcin; ucOCN—undercarboxylated osteocalcin.

**Table 4:** Serum OCN levels across age groups and endocrine states.

Population	Mean OCN Level	Reference Range	Clinical Significance	References
Pediatric patients with short stature (GH-related disorders)	OCN is typically <1.03 ng/mL in GHD (cut-off 1.026 ng/mL for differentiating GHD from ISS)	Not standardized	Reflects reduced bone turnover; high diagnostic value for differentiating GHD vs. ISS	[22]
Healthy pediatric population—reference intervals	80.56 ng/mL (age-dependent)	17.81 ng/mL–191.92 ng/mL	Provides validated pediatric reference intervals across childhood and adolescence	[23]
Healthy pediatric population—normative data	Age-dependent values vary across childhood	Age-specific pediatric ranges reported	Provides normative osteocalcin concentrations for healthy children, enabling comparison with disease states	[24]
Pediatric populations (systematic review and meta-analysis)	Reported tOCN, ucOCN, and cOCN values vary by metabolic status (healthy vs. T1DM vs. T2DM)	Ranges differ across included studies	Identifies osteocalcin–metabolic correlations (glycemia, HbA1c, weight, waist circumference) in >3000 children	[25]
Infancy and early childhood (0–8 years)	Higher levels in infancy; decline with age	Varies by developmental stage	Early OCN levels correlate with later anthropometry and fat-free mass	[26]
Healthy Adults	9–42 ng/mL	3.7–31.7 ng/mL	Marker of bone formation; used in osteoporosis monitoring	[8]
Adults with GH Deficiency	Approximately 18 ng/mL	3.7–31.7 ng/mL	Indicates low bone turnover; rises after GH therapy	[27]
Adults after GH Replacement	up to 62–66 ng/mL	—	Helpful in monitoring anabolic response to GH	[27]

Abbreviations: cOCN—carboxylated osteocalcin; GHD—Growth Hormone Deficiency; GH—Growth Hormone; HbA1c—glycated hemoglobin; ISS—Idiopathic Short Stature; OCN—osteocalcin; T1DM—type 1 diabetes mellitus; T2DM—type 2 diabetes mellitus; tOCN—total osteocalcin; ucOCN — undercarboxylated osteocalcin.

**Table 5:** Clinical Evidence Linking ucOCN to MetS Components.

Population	Key Findings	References
Men aged ≥65 years living independently	Lower ucOCN was associated with increased WC, TG, fasting glucose, and blood pressure, and decreased HDL-C.	[54]
Older adult men (longitudinal cohort)	Higher ucOCN is linked to a lower incidence of diabetes.	[55,61]
Patients T2DM	ucOCN inversely correlated with plasma glucose levels and total fat mass	[56]
Adults with MetS	Lower ucOCN is associated with increased cardiometabolic risk.	[62]

Abbreviations: BP—blood pressure; HDL-C—high-density lipoprotein cholesterol; MetS—metabolic syndrome; T2DM—type 2 diabetes mellitus; TG—triglycerides; ucOCN—undercarboxylated osteocalcin; WC—waist circumference.

**Table 6:** Physiological and pathological conditions associated with plasma OCN concentrations and their clinical relevance.

Condition	OCN Trend	Clinical Relevance	References
Childhood (dynamic bone growth)	Increased	High bone turnover during growth	[5,15,70]
Postmenopausal women	Increased	Increased bone turnover; osteoporosis risk	[5,47,70]
Bone fractures	Increased	Accelerated bone remodeling	[5,15,70]
Osteomalacia	Increased	Defective mineralization; compensatory turnover	[15,70]
Tumor metastases to bone	Increased	Increased bone resorption and formation	[15,70]
Potassium deficiency	Decreased	Possible impaired bone metabolism	[70]
Hypoparathyroidism	Decreased	Low bone turnover	[5,15,70]
Hypothyroidism	Decreased	Reduced metabolic activity; low bone turnover	[5,15,70]
Long-term corticosteroid therapy	Decreased	Suppressed bone formation; osteoporosis risk	[5,15,70]
Liver failure	Decreased	Impaired protein synthesis; low OCN production	[15,67]

**Table 7:** Randomized controlled trials and meta-analysis on vitamin K_2_ (MK-4 and MK-7) and its effects on BMD, OCN carboxylation, and fracture risk in women.

Population	Vitamin K_2_ Form	Dose/Duration	Effect	Reference
Healthy postmenopausal women (n = 244)	MK-7	180 µg/day, 3 years	Slowed loss of lumbar spine and femoral neck BMD; improvement in bone strength indices; ucOCN reduced by approximately 51%; cOCN increased by approximately 21%	[73]
Healthy adults (men and women), dose–response trial (n = 42)	MK-7	10–360 µg/day, 12 weeks	Dose-dependent improvement of extra-hepatic vitamin K status; consistent reduction in ucOC; improved ucOC/cOC ratio	[74]
Postmenopausal women (n = 381)	MK-4	45 mg/day, 12 months	Reduction in ucOCN; no significant effect on BMD	[75]
Postmenopausal women (Meta-analysis of 16 RCTs, n = 6425)	MK-4/ MK-7	45 mg MK-4; 180–375 µg MK-7; 6–36 months	Increased lumbar spine BMD; decreased ucOC; improved ucOC/cOC ratio; reduced fracture risk	[76]
Healthy postmenopausal women (n = 244)—vascular endpoints	MK-7	180 µg/day, 3 years	Significant improvement in arterial stiffness (cfPWV, stiffness index β); dp-ucMGP reduced by 50%, indicating improved vitamin K status	[77]

Abbreviations: BMD—bone mineral density; cfPWV—carotid–femoral pulse wave velocity; cOCN—carboxylated osteocalcin; dp-ucMGP—dephospho undercarboxylated matrix Gla protein; MK-4—menaquinone-4; MK-7—menaquinone-7; OCN—osteocalcin; RCT—randomized controlled trial; ucOCN—undercarboxylated osteocalcin.

**Table 8:** Receptors, Signaling Pathways, and Physiological Outputs.

Receptor/Model	Expression/Target	Core Pathway (s)	Physiological Output/Endpoint	References
GPRC6A	Pancreatic β cells	PI3K/Akt/mTOR	Increased insulin secretion; increased β-cell mass	[11]
GPRC6A	Skeletal muscle	AS160 phosphorylation	Increased GLUT4 translocation; increased glucose uptake	[4]
GPRC6A	Adipocytes	Rap1–CREB signaling	Increased adiponectin; increased thermogenesis	[51]
GPRC6A	Hepatocytes	Nrf2 activation; reduced JNK activity; AMPK–FOXO1/BCL6/CD36 regulation	Reduced hepatic steatosis; reduced ER stress	[11,82,85,86]
GPRC6A	Parasympathetic neurons	Cholinergic modulation	ASR with reduced vagal tone and reduced acetylcholine synthesis	[83]
GPRC6A	Leydig cells	cAMP–PKA–MEK/ERK–CREB	Increased testosterone biosynthesis	[84]
GPR158	Hippocampal neurons	CREB/BDNF signaling	Improved memory; reduced anxiety; enhanced LTP and PPF	[12]
GPR37	Oligodendrocytes	Context-dependent intracellular signaling	Increased myelination; neuroprotection	[13]
Clinical (T2DM)	Human metabolic studies	Improvement of glycemic indices without receptor-specific mechanistic resolution	Decreased glycemia; decreased HOMA-IR	[85]
Postmenopausal women (review-based evidence)	Human adipose-metabolic axis	Adipokine modulation without significant alteration of insulin-signaling pathways	Increased adiponectin; no change in HOMA-IR	[78]

Abbreviations: ACh—acetylcholine; AMPK—AMP-activated protein kinase; AS160—Akt substrate of 160 kDa; ASR—acute stress response; BCL6—B-cell lymphoma 6 protein; CD36—fatty-acid translocase; CREB—cAMP response element-binding protein; ER—endoplasmic reticulum; ERK—extracellular signal-regulated kinase; FOXO1—forkhead box protein O1; GLUT4—glucose transporter type 4; GPR37—G protein-coupled receptor 37; GPR158—G protein-coupled receptor 158; GPRC6A—G protein-coupled receptor class C group 6A; HOMA-IR—homeostatic model assessment of insulin resistance; JNK—c-Jun N-terminal kinase; LTP—long-term potentiation; MEK—MAPK/ERK kinase; mTOR—mechanistic target of rapamycin; Nrf2—nuclear factor erythroid 2-related factor 2; PKA—protein kinase A; PPF—paired-pulse facilitation; Rap1—Ras-related protein 1; T2DM—type 2 diabetes mellitus.

**Table 9:** Core intracellular signaling pathways downstream of GPRC6A.

Signaling Pathway	Functional Effects	References
PI3K–Akt–mTOR pathway	Supports β-cell proliferation, insulin granule formation, and muscle glucose uptake via FOXO1 exclusion and mTORC1-dependent nutrient sensing.	[90]
PLCβ–IP_3_–Ca^2+^ signaling	Catalyzes rapid Ca^2+^ release, enhancing insulin exocytosis and activating Ca^2+^-dependent steroidogenic enzymes in Leydig cells.	[91]
Ras–MEK–ERK cascade	Promotes mitochondrial biogenesis, OXPHOS gene expression, and cell survival under metabolic stress.	[92]
cAMP–PKA axis	Phosphorylates StAR, CYP11A1, CYP17A1, and regulates hepatic redox tone and lipid handling.	[93]
AMPK activation	Increases fatty-acid oxidation and autophagy while suppressing lipogenesis (via decreased SCD1), stabilizing hepatocyte and myocyte energy balance.	[94]
Vesicular trafficking and GLUT4 translocation	ucOCN enhances insulin-stimulated glucose disposal by phosphorylating AS160/TBC1D4, relieving Rab GTPase inhibition, increasing GLUT4 vesicle docking and membrane translocation, and improving mitochondrial coupling during exercise.	[95]
Redox regulation and ER-stress attenuation	ucOCN activates Nrf2 to induce HO-1, NQO1, and glutathione synthesis enzymes, while suppressing JNK signaling and reducing UPR activation (decreased CHOP, decreased XBP1 splicing, decreased ATF4), protecting cells from oxidative, lipotoxic, and inflammatory stress.	[96,97,98]

Abbreviations: Akt—protein kinase B; AMPK—AMP-activated protein kinase; AS160/TBC1D4—Akt substrate of 160 kDa/Tre-2–Bub2–Cdc16 domain family member 4; ATF4—activating transcription factor 4; cAMP—cyclic adenosine monophosphate; CHOP—C/EBP homologous protein; CYP11A1—cytochrome P450 family 11 subfamily A member 1; CYP17A1—cytochrome P450 family 17 subfamily A member 1; ERK—extracellular signal-regulated kinase; GLUT4—glucose transporter type 4; HO-1—heme oxygenase-1; IP_3_—inositol 1,4,5-trisphosphate; JNK—c-Jun N-terminal kinase; MEK—MAPK/ERK kinase; mTORC1—mechanistic Target of Rapamycin Complex 1; NQO1—NAD(P)H quinone dehydrogenase 1; Nrf2—nuclear factor erythroid 2-related factor 2; OXPHOS—oxidative phosphorylation; PI3K—phosphoinositide 3-kinase; PKA—protein kinase A; PLCβ—phospholipase C beta; Rab GTPases—Ras-related small GTP-binding proteins; SCD1—stearoyl-CoA desaturase-1; StAR—steroidogenic acute regulatory protein; UPR—unfolded protein response; XBP1—X-box binding protein 1.

**Table 10:** Key neurobiological pathways modulated by OCN (GPR158 axis).

Pathway/Mechanism	Functional Effect	Relevance to OCN Biology	References
CREB/BDNF signaling	Enhanced LTP, improved memory performance, and reduced anxiety-related behavior	OCN activates GPR158-dependent pathways that converge on CREB phosphorylation, promoting BDNF expression and supporting its neurocognitive effects.	[99]
IRS–PI3K–Akt coupling	Support of astrocytic aerobic glycolysis and improved neurometabolic integration	OCN enhances insulin sensitivity and modulates PI3K–Akt signaling, facilitating neuroenergetic efficiency in the brain.	[100]
Upregulation of TPH2 and TH	Increased synthesis of serotonin, dopamine, and noradrenaline	OCN stimulates monoaminergic neurons, increasing TH and TPH2 expression and thereby improving mood, cognition, and stress resilience.	[101]

Abbreviations: Akt—protein kinase B; BDNF—brain-derived neurotrophic factor; CREB—cAMP-response element binding protein; IRS—insulin receptor substrate; LTP—long-term potentiation; OCN—osteocalcin; PI3K—phosphoinositide 3-kinase; TH—tyrosine hydroxylase; TPH2—tryptophan hydroxylase 2.

**Table 11:** ucOCN–GPR37-related neurobiological pathways and functional effects.

Pathway/Mechanism	Functional Effect	Relevance to OCN Biology	References
ucOCN–GPR37 signaling in oligodendrocytes	Support of myelin integrity and oligodendrocyte maturation	ucOCN binding to GPR37 promotes oligodendrocyte lineage differentiation and contributes to white-matter stability.	[102]
ucOCN–GPR37 activation in dopaminergic circuits	Protection of dopaminergic neurons; maintenance of nigrostriatal function	GPR37 activation enhances dopaminergic resilience and reduces vulnerability to metabolic and oxidative stress.	[102]
GPR37 intracellular signaling (ERK/Akt, Ca^2+^)	Increased stress tolerance; reduced apoptosis; neuroprotection	GPR37-dependent modulation of kinase pathways supports OCN-related neuroprotection.	[103]
GPR37 regulation of neuroinflammation (IL-6 axis)	Reduced neuroinflammatory signaling; protection against degeneration	Oligodendrocyte GPR37 signaling interfaces with IL-6 to regulate neuroinflammation.	[104]

Abbreviations: Akt—protein kinase B; ERK—extracellular signal-regulated kinase; GPR37—G protein–coupled receptor 37; IL-6—interleukin-6; OCN—osteocalcin; OPC—oligodendrocyte precursor cell; ucOCN—undercarboxylated osteocalcin.

**Table 12:** Metabolic Endpoints Linked to ucOCN.

Endpoint	Model	OCN Metric	Main Finding	Interpretation	References
Insulin secretion	Pancreatic β-cells (rodent, *in vitro*)	ucOCN	Increased insulin release via PI3K/Akt/mTOR; hyperglycemia potentiates ucOCN-induced Ca^2+^ influx	ucOCN functions as a β-cell secretagogue; response is glucose-dependent	[86,87]
Insulin sensitivity	*Ocn*^−/−^ and *Esp*^−/−^ mice	ucOCN	Reduced sensitivity in *Ocn*^−/−^; increased sensitivity in *Esp*^−/−^	Bone-derived ucOCN improves insulin action (genetic evidence)	[24]
Adiponectin	Postmenopausal women (RCT)	ucOCN (decreased ~70% after MK-7)	Increased adiponectin; no change in HOMA-IR	MK-7 alters adipokine profile without improving insulin resistance in healthy women.	[96]
Glycemia and HOMA-IR	T2DM patients (RCT, 3 months)	ucOCN/cOCN ratio	Reduced fasting glucose and reduced HOMA-IR under vitamin D_3_ + K_2_	D_3_ + K_2_ improves glycaemia and insulin resistance in short-term trials	[75]
BF%	Observational meta-analyses	ucOCN/tOCN	Inverse association with BF%, BMI, fasting glucose, and HbA1c	Higher ucOCN/tOCN linked to lower adiposity and improved glycemic markers (small effect, high heterogeneity)	[93,94]
Muscle glucose uptake	Mouse myofibers	ucOCN and insulin	Increased GLUT4 translocation and glucose uptake	ucOCN amplifies insulin-stimulated glucose uptake via AS160(TBC1D4)	[91]
Incident T2DM risk	EPIC-NL prospective cohort	tOCN/ucOCN	No association with T2DM incidence	Large-scale human data indicate context-dependent OCN–glucose associations	[95]
Bone density–glucose axis	Mendelian randomization + cohorts	Genetically elevated BMD	Increased T2DM risk and increased 2 h OGTT glucose	Genetic evidence suggests bone–glucose crosstalk beyond OCN alone	[97,98]
Whole-body glucose/insulin regulation	*Bglap*/*Bglap2* dKO (CRISPR) mice	OCN gene deletion	No endocrine abnormalities in OCN-deficient mice	Modern genetic models challenge classical endocrine functions of OCN (possible compensation effects)	[99,100]

Abbreviations: AS160—Akt substrate of 160 kDa (TBC1D4); BMD—bone mineral density; cOCN—carboxylated osteocalcin; BF%—percent body fat; BMI—body mass index; CRISPR—clustered regularly interspaced short palindromic repeats; dKO—double knockout; EPIC-NL—European Prospective Investigation into Cancer and Nutrition, Netherlands cohort; *Esp*^−/−^—mice lacking *Esp*; GLUT4—glucose transporter type 4; HOMA-IR—homeostatic model assessment of insulin resistance; MK-7—menaquinone-7 (vitamin K_2_); OCN—osteocalcin; *Ocn*^−/−^—mice lacking *Ocn*; OGTT—oral glucose tolerance test; PI3K/Akt/mTOR—phosphoinositide 3-kinase/protein kinase B/mechanistic target of rapamycin; RCT—randomized controlled trial; TBC1D4—TBC1 domain family member 4; tOCN—total osteocalcin; ucOCN—undercarboxylated osteocalcin.

**Table 13:** Impact of *Esp* and *Ocn* gene deletion on glucose metabolism in mice.

Mice Phenotype (Genotype)	β-Cell Mass	Insulin Secretion	Insulin Sensitivity	Glucose Tolerance (OGTT)	T2DM Risk	References
*Ocn^−^*/*^−^* (OCN gene KO)	Decreased	Decreased	Decreased	Impaired	Increased	[2,50,93]
*Esp^−^*/*^−^* (ESP gene KO; OST-PTP deficiency)	Increased	Increased	Increased	Enhanced	Decreased	[2,93]
*Bglap*/*Bglap2* dKO (CRISPR-generated)	Similar to WT	Similar to WT	Similar to WT	No impairment (vs. WT)	Similar to WT	[34,96,109]

Abbreviations: *Bglap*/*Bglap2* dKO—*Bglap*/*Bglap2* double knockout; CRISPR—clustered regularly interspaced short palindromic repeats; *Esp*^−/−^—*Esp* knockout mice; KO—knockout; *Ocn*^−/−^—*Ocn* knockout mice; OGTT—oral glucose tolerance test; OST-PTP—osteotesticular protein tyrosine phosphatase; T2DM—type 2 diabetes mellitus; WT—wild type.

**Table 14:** OCN Effects on Glucose Homeostasis and Adiposity: Mechanisms vs. Evidence.

Mechanism/Intervention	Physiological Effect	Evidence Type	References
ucOCN → GPRC6A (β-cells) → PI3K/Akt/mTOR	Increased insulin secretion; increased β-cell proliferation	Genetic and mechanistic	[11,86]
ucOCN → adipocytes → Rap1–ERK/CREB	Increased adiponectin; increased thermogenic/energy-expenditure programming	Preclinical (cells/mice)	[24,90]
ucOCN ↔ fasting glucose/adiposity	Decreased glucose; decreased BMI/BF% (observational correlations)	Observational meta-analyses	[93,94]
MK-7 (375 µg/day for 12 months)	Decreased ucOCN (~70%); increased adiponectin; no change in HOMA-IR	RCT (healthy women)	[96]
Vitamin D_3_ and K_2_, (T2DM, 3 months)	Decreased glycemia; decreased HOMA-IR; increased ucOCN/cOCN ratio	RCT (T2DM)	[74]
ucOCN and insulin (skeletal muscle)	Increased GLUT4 translocation and glucose uptake; AS160(TBC1D4) convergence	Physiology/preclinical	[91,92]
Genetically elevated BMD	Increased T2DM risk and increased 2 h OGTT glucose	Mendelian randomization/cohort	[97,98]

Abbreviations: → denotes a directional mechanistic link; denotes a bidirectional association without established causality. AS160—Akt substrate of 160 kDa (TBC1D4); BF%—percent body fat; BMD—bone mineral density; BMI—body mass index; cOCN—carboxylated osteocalcin; CREB—cAMP response element-binding protein; ERK—extracellular signal-regulated kinase; GLUT4—glucose transporter type 4; GPRC6A—G-protein-coupled receptor family C group 6 member A; HOMA-IR—homeostatic model assessment of insulin resistance; MK-7—menaquinone-7 (vitamin K2); MR—Mendelian randomization; OCN—osteocalcin; OGTT—oral glucose tolerance test (2 h glucose); PI3K/Akt/mTOR—phosphoinositide 3-kinase/protein kinase B/mechanistic target of rapamycin; Rap1—Ras-related protein 1; RCT—randomized controlled trial; T2DM—type 2 diabetes mellitus; TBC1D4—TBC1 domain family member 4; ucOCN—undercarboxylated osteocalcin.

**Table 15:** ucOCN–GLP-1 Axis: Mechanistic Pathways and Evidence Types.

Mechanism	Effect	Evidence Type	Reference
ucOCN → Piezo1 mechanosensitive channel	Increased GLP-1 release under luminal stretch	Experimental	[106]
ucOCN and nutrient combinations (amino acids + FA)	Potentiated GLP-1 release	Preclinical	[107]
ucOCN → GPRC6A on L-cells	Increased GLP-1 secretion	Preclinical (cell/mouse)	[108]

Abbreviations: → denotes signaling through/activation of the indicated receptor or channel; FA—fatty acids; GLP-1—glucagon-like peptide-1; GPRC6A—G protein-coupled receptor class C group 6 member A; L-cells—GLP-1-secreting enteroendocrine L-cells of the intestine; Piezo1—mechanosensitive ion channel Piezo1; ucOCN—undercarboxylated osteocalcin.

**Table 16:** OCN–Muscle Axis: Mechanisms and Outcomes.

Mechanism	Effect	Evidence	References
ucOCN → GPRC6A → AS160	Increased GLUT4 translocation	Mouse myofibers	[4]
ucOCN and contraction	Increased glucose uptake	Ex vivo human muscle	[109]
ucOCN → IL-6	Increased muscle hypertrophy and regeneration	Mouse	[111]

Abbreviations: → denotes a directional mechanistic interaction within the ucOCN–signaling pathway. AS160—Akt substrate of 160 kDa; GPRC6A—G protein-coupled receptor class C group 6 member A; GLUT4—glucose transporter type 4; IL-6—interleukin 6; ucOCN—undercarboxylated osteocalcin.

**Table 17:** Reproductive phenotypes in *Ocn^−/−^*, *Esp^−/−^* and Leydig-specific Gprc6a knockout mice, based on [113].

Genotype/Model	Reproductive Organ Weight	Sperm Count	Leydig Cell Maturation	Serum LH	Conclusion
*Ocn* ^−/−^	Decreased	Decreased	Decreased	Increased	Loss of bone-derived OCN impairs Leydig cell maturation and testosterone secretion despite high LH, consistent with HPG-axis dysregulation.
*Esp* ^−/−^	Increased	Increased	Increased	No change	Removal of the osteoblastic brake on insulin signaling increases ucOCN and improves reproductive parameters.
Leydig-specific *Gprc6a* cKO	Decreased	Decreased	Decreased	Increased	Receptor-level phenocopy of *Ocn*^−/−^; GPRC6A is required for ucOCN-mediated Leydig-cell function.

Abbreviations: cKO—conditional knockout; GPRC6A—G protein-coupled receptor class C group 6A; HPG–hypothalamus–pituitary–gonadal axis; LH—luteinizing hormone; OCN—osteocalcin; ucOCN—undercarboxylated osteocalcin. All phenotypic changes are reported relative to wild-type controls.

**Table 18:** Osteocalcin–brain mechanisms, effects, and contexts.

Mechanism/Pathway	Effect	Clinical Context/Model	References
OCN → GPR158 → CREB–BDNF	Increased neurogenesis; increased memory; reduced anxiety-like behavior	Hippocampus (CA3), mice	[12]
OCN → IRS→PI3K–Akt coupling	Neurometabolic support (astrocytic aerobic glycolysis) for plasticity	Hippocampus/astrocytes, mice	[12]
OCN → increased tryptophan hydroxylase 2; increased tyrosine hydroxylase	Increased serotonin, increased dopamine, increased noradrenaline	Depression models, mice	[3]
OCN → decreased Gad1/Gad2	Reduced GABAergic tone	Depression/stress models, mice	[3]
OCN → increased BDNF signaling	Increased synaptic plasticity	Alzheimer’s/aging (preclinical)	[12]
OCN → increased autophagy in hippocampus	Reversal of age-related memory decline	Alzheimer’s (preclinical)	[122]
OCN → GPR37 signaling in oligodendrocytes/dopaminergic neurons	Myelin- and dopamine-related pathways; context-dependent motor outcomes	Parkinson’s disease (preclinical)	[13]
OCN → decreased PHD1 → increased PPP	Reduced pyroptosis; increased neuronal survival	Ischemic stroke	[123]
Maternal OCN → placental transfer	Reduced neuronal apoptosis; normal brain development	Prenatal neurogenesis	[3]

Abbreviations: → denotes a sequential signaling step (ligand–receptor activation or downstream effector engagement). Akt—protein kinase B; BDNF—brain-derived neurotrophic factor; CREB—cAMP response element-binding protein; Gad1—glutamate decarboxylase 1; Gad2—glutamate decarboxylase 2; GPR158—G protein-coupled receptor 158; GPR37—G protein-coupled receptor 37; IRS—insulin receptor substrate; OCN—osteocalcin; PHD1—prolyl hydroxylase domain-containing protein 1; PI3K—phosphoinositide 3-kinase; PPP—pentose phosphate pathway. Findings derive primarily from preclinical mouse or cell models; human-level insights are summarized in the main text.

**Table 19:** Emerging mechanisms of osteocalcin in neurodegeneration and stress.

Mechanism (Emerging)	Effect	Disease/Model	References
Network-level modulation in regions with high GPR37/GPR158 expression (pharmacology, together with resting-state fMRI)	Regional activity shifts consistent with OCN-responsive circuits	Mouse brain (preclinical)	[124]
UPR suppression (HSPA5/XBP1/CHOP)	Increased neuronal survival under ER stress	Neurodegeneration models (preclinical)	[125]
Epigenetic repression of GPR158 under hyperglycemia	Decreased GPR158 expression; bone–brain decoupling	Diabetes, ageing (preclinical)	[126]

Abbreviations: BLA—basolateral amygdala; CHOP—C/EBP homologous protein; ER—endoplasmic reticulum; fMRI—functional magnetic resonance imaging; GPR37—G protein-coupled receptor 37; GPR158—G protein-coupled receptor 158; HSPA5—Heat Shock Protein Family A Member 5; OCN—osteocalcin; UPR—unfolded protein response; XBP1—X-box binding protein 1.

**Table 20:** Osteocalcin in acute stress response: models, endpoints, and mechanisms.

Endpoint (ASR Feature)	Model/Subjects	OCN Metric	Key Finding	Mechanistic Note	References
Rapid surge of bioactive OCN	Mice, rats, humans (public speaking/psychosocial stress)	ucOCN (bioactive form)	Minute-scale increase: approximately 50–150% in rodents; rise in humans during public speaking	Stress triggers osteoblast glutamate uptake, thereby reducing OCN inactivation and increasing its release. Bioactive OCN is required for the acute stress response (ASR)	[127,128]
Autonomic rebalancing (reduced vagal tone; sympathetic response unmasked)	Rodents; adrenalectomized animals	ucOCN (bioactive form)	OCN inhibits ACh synthesis and release in post-ganglionic parasympathetic neurons; ASR persists without adrenal glands	OCN acts directly on parasympathetic neurons to decrease vagal output	[127,128]
Adrenal/HPA interaction (developmental capacity)	Rodents; primates	Total OCN and/or ucOCN (study-dependent measurement)	OCN promotes adrenal growth and steroidogenesis; loss of signaling blunts corticosterone response during ASR	Embryonic OCN establishes lifelong adrenal capacity (SF-1/MC2R/CYP11B1/2 regulation)	[129]
Anti-inflammatory modulation	Preclinical macrophage and immune models; rodent stress studies	OCN not directly quantified	OCN restrains pro-inflammatory signaling and modulates phagocytic activity	Context-dependent NF-κB attenuation	[136]
Exercise as a physiological stressor (muscle–bone–muscle axis)	Adults; rodent–human translation	Total OCN or ucOCN (depending on protocol)	Acute aerobic exercise increases circulating OCN. Muscle-derived IL-6 stimulates osteoblasts to release OCN, forming a feed-forward loop that enhances exercise capacity	Muscle-derived IL-6 stimulates osteoblast-dependent OCN release; OCN improves muscle fuel utilization.	[134,135]
Human stress paradigm (TSST)	Healthy adults (TSST)	OCN not measured in these studies	TSST reliably increases BP, HR, anxiety, and HPA output, producing minute-scale endocrine responses	Standardized social-evaluation stressor with robust multisystem effects	[131,132,133]
Adrenal insufficiency—preserved ASR via OCN	Patients with adrenal insufficiency, adrenalectomized rodents	Total OCN or ucOCN (model-dependent measurement)	Despite impaired adrenal function, ASR remains intact and is associated with increased circulating OCN	OCN provides adrenal-independent endocrine mediation of the ASR	[127,128]
Amygdala-to-bone signaling in ASR	Mice (BLA manipulation)	ucOCN release (experimentally measured)	Chemogenetic inhibition of the BLA alters OCN release and modifies ASR outcomes	A brain-to-bone glutamatergic pathway regulates osteoblast-derived OCN release	[127,128]
Biomarker and translational outlook	Narrative reviews	OCN not systematically quantified	OCN proposed as a biomarker of stress resilience and as a potential therapeutic target in stress-related metabolic and neuropsychiatric states	Integrates autonomic, adrenal, and immune axes; clinical translation under development	[136,137]

Abbreviations: ACh—acetylcholine; ASR—acute stress response; BLA—basolateral amygdala; BP—blood pressure; CHOP—C/EBP homologous protein; CYP11b1/2—cytochrome P450 family 11 subfamily B (steroidogenic enzymes); HPA—hypothalamic–pituitary–adrenal axis; HR—heart rate; IL-6—interleukin-6; MC2R—melanocortin 2 receptor; NF-κB—nuclear factor kappa B; OCN—osteocalcin; SF1—steroidogenic factor-1; TSST—Trier Social Stress Test; ucOCN—undercarboxylated osteocalcin.

**Table 21:** Osteocalcin-based mechanisms relevant to NAFLD: hepatic signalling and enterohepatic crosstalk.

Strategy/Form	Core Mechanism(s) in the NAFLD Axis	Evidence	References
ucOCN (native hormone)	ucOCN engages GPRC6A-dependent hepatic signaling, activating Nrf2 and suppressing JNK to buffer oxidative and ER stress, while AMPK-driven repression of SCD1 curbs *de novo* lipogenesis	Mouse NAFLD models; hepatocyte studies	[138,139,141]
csOCN (synthetic peptide)	AMPK activates FOXO1/BCL6 transcriptional program that represses CD36; while direct csOCN-CD36 docking and colocalization further limit hepatocellular FA uptake	Translational preclinical (oral dosing in NAFLD mice)	[140]
Enterohepatic incretin arm	ucOCN induces GLP-1 from L-cells; GLP-1R signaling is required for OCN’s metabolic benefits; Piezo1 in L-cells strengthens GLP-1 release under mechanical cues	In vivo mouse; L-cell paradigms	[143,144,145]
Human clinical signal	Lower serum OCN shows an inverse association with the severity of steatosis/fibrosis; higher NAFLD incidence and lower remission (sex-specific)	Two cohorts and animal models	[142]

Abbreviations: AMPK—AMP-activated protein kinase; CD36—fatty acid translocase; csOCN—chemically synthesized osteocalcin; FA—fatty acids; FOXO—forkhead box protein O1; GLP-1—glucagon-like peptide 1; GLP-1R—GLP-1 receptor; GPRC6A—G protein-coupled receptor, class C group 6A; JNK—c-Jun N-terminal kinase; L-cells—GLP–1-secreting enteroendocrine L-cells of the intestine; Nrf2—nuclear factor erythroid 2-related factor 2; Piezo1—mechanosensitive ion channel Piezo1; SCD1—stearoyl-CoA desaturase 1; ucOCN—undercarboxylated osteocalcin.

**Table 22:** Bone turnover and vitamin K-dependent biomarkers in CKD (G3–G5D): analytic caveats, clinical use, and suggested monitoring.

Marker	Kidney Dependence/Clearance	Primary Clinical Use in CKD-MBD	Strengths	Limitations in CKD	Suggested Monitoring	References
PTH (intact)	Not primarily renally cleared	Turnover status (high vs. low), guide ROD phenotype, and therapy	Widely available; KDIGO-endorsed	Episodic variability; assay heterogeneity	G3: every 6–12 months; G4: every6–12 months; G5/G5D: every 3–6 months; ↑frequency with therapy changes	[147,148]
BALP (bone-specific ALP)	Renally independent	Bone formation; differentiate high vs. adynamic turnover	Correlates with bone biopsy; automation available	Confounding by cholestasis (total ALP); ensure bone-specific assay	Same cadence as PTH; adjust to treatment	[14,151]
Intact PINP	Minimal renal effect (intact/trimeric assay)	Bone formation monitoring and therapy response	Preferred vs. total PINP in CKD; standardized	Availability varies; cost	Baseline, then every 3–6 months under antiresorptives/anabolics	[5,14,151]
TRACP-5b	Renally independent	Bone resorption; complement to BALP and PTH	Not affected by GFR	Limited automation in some labs	Baseline and every 6–12 months; tighter under therapy	[14,151]
β-CTX-I	Renally cleared; accumulation with reduced clearance	Resorption (general population)	Well-established outside CKD	Unreliable in advanced CKD; avoid for turnover in G4–G5D	Not recommended beyond G3	[5,14,151]
OCN (total/N-MID)	Renally cleared (intact & fragments)	Adjunct turnover marker with PTH and BALP	N-MID: preanalytical stability	Clearance confounding; circadian variation	Baseline and every 6–12 months if used; always interpret with PTH/BALP	[8,14]
ucOC/%ucOC	Reflects vitamin K status (carboxylation), not renal clearance	Functional K status; pediatric fracture signal	Sensitive to K; responsive to MK-7	Assay heterogeneity; endpoints inconsistent	Baseline and after diet/supplement trials (6–12 weeks)	[152,153,154,157]
dp-ucMGP	VKDP biomarker	Complementary K-status readout	Strongly responsive to K; calcification biology	Endpoint uncertainty; assay variability	Baseline and post-intervention (6–12 weeks)	[153,156]

Abbreviations: ALP—alkaline phosphatase; BALP—bone-specific alkaline phosphatase; β-CTX-I—β-isomerized C-terminal telopeptide of type I collagen; dp-ucMGP—dephospho-undercarboxylated matrix Gla protein; G3–G5D denote CKD stages as defined by KDIGO (G3: estimated glomerular filtration rate (eGFR) 30–59 mL/min/1.73 m^2^; G4: 15–29 mL/min/1.73 m^2^; G5: <15 mL/min/1.73 m^2^; G5D: stage 5 on dialysis); GFR—glomerular filtration rate; MK-7—menaquinone-7; N-MID—N-terminal/mid-region osteocalcin fragment; OCN—osteocalcin; PINP—procollagen type I N-terminal propeptide; PTH—parathyroid hormone; TRACP-5b—tartrate-resistant acid phosphatase isoform 5b; ucOC—undercarboxylated osteocalcin; VKDP—vitamin K-dependent proteins.

**Table 23:** Key molecular steps reinforcing the mediator mechanism of ucOCN in vascular cells.

Step/Process	Effect/Outcome	References
ucOCN binds to the IRβ receptor	Activates IRS-1 and initiates PI3K/Akt signaling	[159]
IRS-1 → PI3K → AKT	Core intracellular cascade	[163]
Endothelial cells: AKT → eNOS	Improved endothelial function and vasodilation	[159]
Smooth muscle: AKT → decreased NF-κB and mTOR signaling	Reduced ER stress, apoptosis, and autophagy	[159]
NO acts on vascular smooth muscle	Maintains vasodilation and vascular tone	[159]
Overall effect	Enhanced vascular homeostasis and metabolic profile	[163]

Abbreviations: → denotes a sequential signaling step (activation of the next component in the pathway). Akt—protein kinase B; eNOS—endothelial nitric oxide synthase; ER—endoplasmic reticulum; IRβ—insulin receptor β-subunit; IRS-1—insulin receptor substrate-1; mTOR—mechanistic target of rapamycin; PI3K—phosphoinositide 3-kinase; NF-κB—nuclear factor kappa B; NO—nitric oxide.

**Table 24:** Dietary Patterns and Osteocalcin Context—Comparative Evidence.

Dietary Pattern	Vitamin K_2_ Intake	% ucOCN Status	Lipid Profile	Inflammatory Markers	Homocysteine	Lp (a)	Vascular Implication	References
Vegan	Very low (MK-7 scarce)	Higher %ucOCN (due to low K_2_ intake)	Lower TC, lower LDL-C, and lower HDL-C compared with other dietary patterns	Low IL-6; moderate TNF-α	Increased (if vitamin B12 is insufficient)	Individually variable (genetically determined); may increase with a poor lifestyle	Potential calcification risk if K_2_ deficiency persists	[180,181,182]
Vegetarian	Low (higher than vegan)	Moderately increased %ucOCN	Lower TC and higher HDL-C compared with vegans	Moderate IL-6	Slightly increased	Individually variable (genetically determined)	Similar pattern to vegan, less pronounced	[180,181]
Pescatarian	Moderate (fish + fermented foods)	Near-normal %ucOCN	Favorable lipid profile; HDL-C highest among all dietary patterns	Moderate IL-6	Within normal range	Typically lower than in vegans (genetically determined)	Protective against calcification	[181,182]
Omnivore	Adequate MK-4 (animal foods) + MK-7 (fermented foods)	Within the expected physiological range	Higher TC and higher LDL-C compared with pescatarians; HDL-C moderate	Highest IL-6; increased hsCRP	Within normal range	Genetically determined; little to no dietary influence	Higher baseline atherogenic risk	[181,182]

Abbreviations: %ucOCN—percentage of undercarboxylated osteocalcin; HDL-C—high-density lipoprotein cholesterol; hsCRP—high-sensitivity C-reactive protein; IL-6—interleukin-6; LDL-C—low-density lipoprotein cholesterol; MK-4/MK-7—menaquinone-4/-7; TC—total cholesterol; TNF-α—tumor necrosis factor-alpha.

**Table 25:** Vascular endpoints linked to OCN.

Endpoint	Model	OCN Metric	Main Finding	Interpretation	References
Endothelial function	Rabbit aorta; HAEC cells (*ex vivo* and *in vitro* studies)	ucOCN 10–30 ng/mL	No adverse effect	ucOCN neutral at physiological levels	[160]
Coronary artery disease severity	Angiographic cohort (human; n = 59)	OCN^+^ EPCs; tOC/ucOCN/cOC	Mixed correlation	OCN^+^ EPCs may reflect calcific shift	[161]
Vascular calcification risk	Genetic association study (human cohort)	Circulating OCN	Associated with calcification markers	OCN may mark vascular risk	[163]
NO bioavailability	Endothelial cells (*in vitro*)	ucOCN → IRβ → PI3K → Akt → eNOS	Increased NO production	ucOCN improves endothelial tone	[159]
ER stress and apoptosis	VSMC culture (*in vitro*)	ucOCN → AKT → decreased NF-κB/mTOR	Reduced ER stress and reduced apoptosis	ucOCN protective under stress	[159]

Abbreviations: → denotes a sequential signaling step (activation or downstream propagation within the pathway). Akt—protein kinase B; cOC—carboxylated osteocalcin; EPC—endothelial progenitor cell; OCN^+^ EPCs—osteocalcin-positive endothelial progenitor cells; eNOS—endothelial nitric oxide synthase; ER—endoplasmic reticulum; HAEC—human aortic endothelial cell; IRβ—insulin receptor β-subunit; mTOR—mechanistic target of rapamycin; NFκB—nuclear factor kappa B; NO—nitric oxide; PI3K—phosphoinositide 3-kinase; tOC—total osteocalcin; ucOCN—undercarboxylated osteocalcin; VSMC—vascular smooth muscle cell.

**Table 26:** OCN roles in oncology—compartment-aware concept map (mediator vs. marker).

Axis	Compartment/Readout	Core Mechanism/Exemplar	Clinical Implication	References
Mediator	Circulating ucOCN; receptor context (GPRC6A; TGF-β/SMAD3)	Prostate cancer: ucOCN activates GPRC6A, engaging ERK, Akt, and mTOR signaling. TNBC: ucOCN signals through the TGF-β/SMAD3 axis, promoting EMT and osteolytic programs.	Receptor-based or pathway-based stratification for targeted therapy	[187,188,189]
Marker	OCN^+^ EPCs; lesional OCN (IHC)	Osteogenic drift in the bone microenvironment; PMN conditioning	Bone metastasis risk stratification; early disease monitoring	[190,191,192,193]

Abbreviations: Akt—protein kinase B; GPRC6A—G protein-coupled receptor class C group 6A; IHC—immunohistochemistry; mTOR—mechanistic target of rapamycin; OCN—osteocalcin; OCN^+^ EPCs—osteocalcin-positive endothelial progenitor cells; PMN—pre-metastatic niche; SMAD3—mothers against decapentaplegic homolog 3; TGF-β—transforming growth factor-β; TNBC—triple-negative breast cancer; ucOCN—undercarboxylated osteocalcin.

**Table 27:** OCN/ucOCN as mediator—receptors, pathways, and phenotypes in prostate cancer and TNBC.

Tumour Type	Compartment/Receptor	Core Pathway (s)	Proximal Phenotype (s)	References
Prostate cancer	GPRC6A on tumor cells	ERK/Akt/mTOR	Increased proliferation; increased mTORC1 readouts; xenograft growth	[187,188]
TNBC	TGF-β/SMAD3 axis	TGF β/SMAD3 (EMT, osteolytic signaling)	Increased EMT; increased MMPs; increased VEGF; bone-tropic signaling	[189]

Abbreviations: Akt—protein kinase B; EMT—epithelial–mesenchymal transition; ERK—extracellular signal-regulated kinase; GPRC6A—G protein-coupled receptor family C group 6 member A; MMPs—matrix metalloproteinases; mTOR—mechanistic target of rapamycin; mTORC1—mechanistic target of rapamycin complex 1; SMAD3—mothers against decapentaplegic homolog 3; TGF-β—transforming growth factor-β; TNBC—triple-negative breast cancer; VEGF—vascular endothelial growth factor.

**Table 28:** OCN as a marker—OCN-positive circulating cells and lesional OCN in bone-tropic diseases.

Readout	Setting/Method	Signal/Utility	Clinical Note	References
OCN^+^ circulating EPCs	Breast cancer; flow cytometry/translational cohorts	Increased OCN^+^ EPCs with bone involvement; useful for early risk discrimination	Candidate biomarker for early bone-metastasis monitoring; requires prospective validation	[190]
Lesional OCN (IHC)	Bone microenvironment; spatial profiling	Osteogenic drift within the PMN; bone-tropic priming	Valuable contextual marker within multiparametric biomarker panels	[191,192,193]
EV signaling	Bone cancer/metastasis	Tumor-derived EVs orchestrate PMN formation and skeletal colonization	Mechanistic framework for niche-aware OCN-based biomarkers	[194,195]

Abbreviations: EV—extracellular vesicles; EPCs—endothelial progenitor cells; IHC—immunohistochemistry; OCN—osteocalcin; OCN^+^ EPCs—osteocalcin-positive endothelial progenitor cells; PMN—pre-metastatic niche.

**Table 29:** Study-design checklist for bone-prone malignancies.

Dimension	Endocrine Arm (ucOCN)	Osteogenic Arm (cOCN/Lesional OCN)	Imaging/Endpoints	Notes
Baseline phenotype	Bioactive ucOCN assay; document vitamin K status	cOCN^+^ EPCs; lesional OCN (IHC if available)	BM presence; BM-PFS	Assay specification; sampling windows
On-therapy dynamics	Fixed-window ucOCN repeats	Matched-time cOCN repeats	Serial BM-PFS; symptoms	Harmonize compartments and timepoints
Mechanistic layer	Receptors/pathways (GPRC6A; TGF-β/SMAD3)	EV panels, if available	Integrate with outcomes	Orthogonal pathway validation

Abbreviations: BM—bone metastasis; BM-PFS—bone metastasis progression-free survival; cOCN—carboxylated osteocalcin; EV—extracellular vesicles; GPRC6A—G protein-coupled receptor class C group 6 member A; IHC—immunohistochemistry; SMAD3—mothers against decapentaplegic homolog 3; TGF-β—transforming growth factor-β; ucOCN—undercarboxylated osteocalcin.

**Table 30:** OCN-centric and adjacent translational hooks across tumor types.

Tumor Type	OCN-Centric Mechanism	Translational Hook	References
Prostate cancer	OCN/ucOCN activates GPRC6A and downstream ERK, Akt, and mTOR pathways	Receptor-based stratification; GPRC6A-axis modulators integrated with AR-directed therapy	[187,188,199]
TNBC	ucOCN signals through the TGF-β/SMAD3 axis (EMT, osteolysis)	SMAD3 modulation; monitoring OCN^+^ EPCs in bone-tropic settings	[189]
Breast cancer (BM risk)	OCN^+^ circulating EPCs as an early BM risk marker	Early bone-targeted strategies require prospective validation	[190]
TNBC (adjacent biology)	Osteoclast-derived glutamine drives GPX4/ATF4-linked PARPi resistance.	PARP-inhibitor and microenvironment-aware co-therapy	[200]
Prostate (osteoblastic lesions)	PSA–osteoblast crosstalk in sclerotic microenvironments	Integrating niche-modifying strategies with AR-targeted therapy	[199]

Abbreviations: Akt—protein kinase B; AR—androgen receptor; ATF4—activating transcription factor 4; BM—bone metastasis; EMT—epithelial–mesenchymal transition; ERK—extracellular signal-regulated kinase; GPX4—glutathione peroxidase 4; GPRC6A—G protein-coupled receptor class C group 6 member A; mTOR—mechanistic target of rapamycin; OCN—osteocalcin; OCN^+^ EPCs—osteocalcin-positive endothelial progenitor cells; PARPi—PARP inhibitor; PSA—prostate-specific antigen; SMAD3—mothers against decapentaplegic homolog 3; TGF-β—transforming growth factor-β; TNBC—triple-negative breast cancer; ucOCN—undercarboxylated osteocalcin.

**Table 31:** Modulators of the OCN Axis: Dietary, Endogenous, and Lifestyle Factors.

Modulator/Source	Dominant Mechanism (s)	Expected Effect on OCN/Bone	References
Vitamin K_2_ (MK-7) from fermented	Cofactor for GGCX → γ-carboxylation	Decreased ucOCN; increased cOCN; supports mineral binding and bone quality	[69,73]
Vitamin D_3_ (diet/sunlight)	Increases BGLAP transcription; promotes osteoblast maturation	Increased OCN synthesis; synergy with K_2_ for functional activation	[66,76]
Polyphenol-rich diet (Mediterranean pattern)	Antioxidant and anti-inflammatory milieu; endothelial support	Indirect support of osteoblast function and vascular health	[69,201]
Microbiome (*Bacillus*, *Bifidobacterium*)	Menaquinone (MK-n) synthesis	Endogenous K_2_ supports OCN carboxylation potential	[65]
Vitamin K insufficiency	Reduced cofactor availability for GGCX	Increased ucOCN; associated with fracture risk	[19,67]
Physical activity/exercise	OCN–muscle crosstalk; metabolic adaptations	Systemic metabolic benefits; OCN engagement	[4]

Abbreviations: BGLAP—bone gamma-carboxyglutamate protein; cOCN—carboxylated osteocalcin; GGCX—γ-glutamyl carboxylase; MK-n—menaquinones containing n isoprenoid units; MK-7—menaquinone 7; NO—nitric oxide; OCN—osteocalcin; ucOCN—undercarboxylated osteocalcin.

**Table 32:** Probiotics and microbiome-directed strategies that may support the K2–OCN axis (selected examples).

Probiotic/Approach	Primary Rationale	Expected Readouts	References
*Bacillus* (e.g., *B. subtilis*)	Spore-forming genus; menaquinone (MK-n) biosynthesis; GI survival; alignment with fermented MK-7 food sources	Reduced dp-ucMGP; increased OCN carboxylation potential (cOCN); maintenance of BMD with dietary synergy	[54,189]
*LactoBacillus*/*LacticaseiBacillus*	Gut–bone immunometabolic modulation (barrier integrity, SCFA production, cytokine tone)	Modestly increased lumbar-spine BMD over 6–12 months; variable effects at the hip; heterogeneous BTMs	[188]
*Bifidobacterium*	SCFA output; epithelial-barrier reinforcement; synergy with fiber and polyphenols	Maintenance of BMD; improved milieu for activation of VKDPs	[55,56]

Abbreviations: BMD—bone mineral density; BTMs—bone turnover markers; cOCN—carboxylated osteocalcin; dp-ucMGP—dephospho-undercarboxylated matrix Gla protein; GI—gastrointestinal; MK-7—menaquinone 7; MK-n—menaquinones containing n isoprenoid units; OCN—osteocalcin; SCFAs—short-chain fatty acids; VKDP—vitamin-K-dependent proteins.

## Data Availability

No new data were created or analyzed in this study. Data sharing is not applicable to this article.

## Abbreviations

Ach	acetylcholine
AGEs	advanced glycation end products
AGOEs	advanced glycoxidation end products
Akt/PKB	protein kinase B
ALP	alkaline phosphatase
AMPK	AMP-activated protein kinase
AS160	Akt substrate of 160 kDa
ASR	acute stress response
ATF4	activating transcription factor 4
ATGL	adipose triglyceride lipase
β-cell	pancreatic beta cell
β-CTX-I	beta–C-terminal telopeptide of type I collagen
BALP	bone-specific alkaline phosphatase
BCL6	B cell lymphoma 6 protein
BDNF	brain-derived neurotrophic factor
BF%	percent body fat
BGLAP	bone gamma-carboxyglutamate protein
BLA	basolateral amygdala
BM	bone metastasis
BMD	bone mineral density
BMI	body mass index
BMS	bone–muscle signaling
BMR	basal metabolic rate
CAC	coronary artery calcification
cAMP	cyclic adenosine monophosphate
cfPWV	carotid–femoral pulse wave velocity
cOCN	carboxylated osteocalcin
CD36	fatty acid translocase
CHOP	C/EBP homologous protein
CIMT	carotid intima–media thickness
CKD	chronic kidney disease
CKD MBD	chronic kidney disease–mineral and bone disorder
CML	carboxymethyl-lysine
CREB	cAMP-response element binding protein
CRISPR	clustered regularly interspaced short palindromic repeats
csOCN	chemically synthesized osteocalcin
CTX-I	C-terminal telopeptide of type I collagen
CYP11A1	cytochrome P450 family 11 subfamily A member 1
CYP17A1	cytochrome P450 family 17 subfamily A member 1
ΔΨ	mitochondrial membrane potential
DI	disposition index
dp-ucMGP	dephospho undercarboxylated matrix Gla protein
DMT1	divalent metal transporter 1
DNA	deoxyribonucleic acid
eGFR	estimated glomerular filtration rate
ECLIA	electrochemiluminescence immunoassay
EGFR	epidermal growth factor receptor
EIA	enzyme immunoassay
ELISA	enzyme-linked immunosorbent assay
EMT	epithelial–mesenchymal transition
ER	endoplasmic reticulum
ERK	extracellular signal-regulated kinase
EPCs	endothelial progenitor cells
EPIC-NL	European Prospective Investigation into Cancer and Nutrition, Netherlands cohort
EPIDOS	Epidemiology of Osteoporosis Study
ESCEO	European Society for Clinical and Economic Aspects of Osteoporosis
FA	fatty acids
FMD	flow-mediated dilation
FOXO1	forkhead box protein O1
GABA	gamma aminobutyric acid
GGCX	γ-glutamyl carboxylase
GH	growth hormone
GLP-1	glucagon-like peptide 1
GLP-1R	glucagon-like peptide 1 receptor
GLUT4	glucose transporter type 4
Gla	γ-carboxyglutamic acid
Glu-OC	glutamic acid osteocalcin epitope
GPR	osteocalcin receptors (G protein-coupled receptors)
GPR37	G protein-coupled receptor 37
GPR158	G protein-coupled receptor 158
GPRC6A	G protein-coupled receptor, class C, group 6 member A
GFR	glomerular filtration rate
GPX4	glutathione peroxidase 4
HbA1c	glycated hemoglobin
HDL-C	high-density lipoprotein cholesterol
HEC	hyperinsulinemic–euglycemic clamp
HIF-1α	hypoxia inducible factor 1 alpha
HOMA IR	homeostatic model assessment of insulin resistance
HPG axis	hypothalamic–pituitary–gonadal axis
HPA axis	hypothalamic–pituitary–adrenal axis
HR	heart rate
hsCRP	high-sensitivity C-reactive protein
HSD3B	3β hydroxysteroid dehydrogenase
HSL	hormone-sensitive lipase
IGF-1	Insulin-Like Growth Factor 1
IHC	immunohistochemistry
IL-6	interleukin 6
IRβ	insulin receptor β subunit
IRS 1	insulin receptor substrate 1
JNK	c Jun N-terminal kinase
L-cells	GLP-1-secreting enteroendocrine L-cells of the intestine
LC–MS	liquid chromatography–mass spectrometry
LDL-C	low-density lipoprotein cholesterol
LH	luteinizing hormone
LOX	lysyl oxidase
Lp(a)	lipoprotein(a)
LTP	long-term potentiation
MC2R	melanocortin 2 receptor
MEK	MAPK/ERK kinase (MAP2K; MAPKK)
MGP	matrix Gla protein
MK-n	menaquinones containing n isoprenoid units
MK 4/MK 7	menaquinone 4/menaquinone 7 (vitamin K2 forms)
MMPs	matrix metalloproteinases
mTOR/mTORC1	mechanistic target of rapamycin (complex 1)
NADPH	nicotinamide adenine dinucleotide phosphate ()
NAFLD	non-alcoholic fatty liver disease
NF-κB	nuclear factor kappa-light-chain-enhancer of activated B cells
NLF	non-linear fitting
N-MID	N terminal/mid region osteocalcin fragment
NO	nitric oxide
NQO1	NAD(P)H quinone dehydrogenase 1
Nrf2	nuclear factor erythroid 2-related factor 2
OCN	osteocalcin
OCN^+^ EPCs	osteocalcin-positive endothelial progenitor cells
OGTT	oral glucose tolerance test
OPN	osteopontin
OXPHOS	oxidative phosphorylation
PARPi	poly(ADP ribose) polymerase inhibitor
PGE2	prostaglandin E2
PHD1	prolyl hydroxylase domain-containing protein 1
PI3K	phosphoinositide 3 kinase
PINP	procollagen type I N-terminal propeptide
PKA	protein kinase A
PLCβ	phospholipase C beta
PMN	pre-metastatic niche
PNPLA2	patatin-like phospholipase domain-containing protein 2
PPF	paired pulse facilitation
PPP	pentose phosphate pathway
PWV	pulse wave velocity
Rab GTPases	Ras-related small GTP-binding proteins
Rap1	Ras-related protein 1
RCT	randomized controlled trial
ROS	reactive oxygen species
PXR	pregnane X receptor
RUNX2	runt-related transcription factor 2
SCFAs	short-chain fatty acids
SCD1	stearoyl CoA desaturase 1
SMAD3	mothers against decapentaplegic homolog 3
SNAP25	synaptosomal-associated protein 25 kDa
SREBP1c	sterol regulatory element binding protein 1c
StAR	steroidogenic acute regulatory protein
SX	steroid and xenobiotic receptor
T1DM	type 1 diabetes mellitus
T2DM	type 2 diabetes mellitus
TBC1D4	domain family member 4
TBS	trabecular bone score
TC	total cholesterol
TGF-β	transforming growth factor-β
TIMP-1	tissue inhibitor of metalloproteinases-1
TNBC	triple negative breast cancer
TNF α	tumor necrosis factor alpha
TRACP 5b	tartrate-resistant acid phosphatase 5b
TSST	Trier Social Stress Test
ucOCN	undercarboxylated osteocalcin
UPR	unfolded protein response
vEPCs	vascular endothelial progenitor cells
VEGF	vascular endothelial growth factor
VFT domain	Venus flytrap domain (of GPRC6A receptor)
VKDP	vitamin K-dependent proteins
VKOR	vitamin K epoxide reductase
VSMC	vascular smooth muscle cell
XBP1	X-box binding protein 1
